# A Shared Transcriptional Identity for Forebrain and Dentate Gyrus Neural Stem Cells from Embryogenesis to Adulthood

**DOI:** 10.1523/ENEURO.0271-21.2021

**Published:** 2022-02-01

**Authors:** Michael J. Borrett, Brendan T. Innes, Nareh Tahmasian, Gary D. Bader, David R. Kaplan, Freda D. Miller

**Affiliations:** 1 Program in Neuroscience and Mental Health, Hospital for Sick Children, Toronto, Ontario M5G 1L7, Canada; 2 Institute of Medical Science, University of Toronto, Toronto, Ontario M5G 1A8, Canada; 3 The Donnelly Centre, University of Toronto, Toronto, Ontario M5G 1A8, Canada; 4 Department of Molecular Genetics, University of Toronto, Toronto, Ontario M5G 1A8, Canada; 5 Department of Physiology, University of Toronto, Toronto, Ontario M5G 1A8, Canada; 6 Michael Smith Laboratories, University of British Columbia, Vancouver, British Columbia V6T 1Z4, Canada; 7 Department of Medical Genetics, University of British Columbia, Vancouver, British Columbia V6T 1Z4, Canada

**Keywords:** forebrain neural stem cells, neurodevelopment, single-cell RNA-sequencing

## Abstract

Adult neural stem cells (NSCs) reside in two distinct niches in the mammalian brain, the ventricular-subventricular zone (V-SVZ) of the forebrain lateral ventricles and the subgranular zone (SGZ) of the hippocampal dentate gyrus. They are thought to be molecularly distinct since V-SVZ NSCs produce inhibitory olfactory bulb (OB) interneurons and SGZ NSCs excitatory dentate granule neurons. Here, we have asked whether this is so by directly comparing V-SVZ and SGZ NSCs from embryogenesis to adulthood using single-cell transcriptional data. We show that the embryonic radial glial precursor (RP) parents of these two NSC populations are very similar, but differentially express a small cohort of genes involved in glutamatergic versus GABAergic neurogenesis. These different RPs then undergo a similar gradual transition to a dormant adult NSC state over the first three postnatal weeks. This dormancy state involves transcriptional shutdown of genes that maintain an active, proliferative, prodifferentiation state and induction of genes involved in sensing and regulating their niche environment. Moreover, when reactivated to generate adult-born progeny, both populations reacquire a development-like state and re-express proneurogenic genes. Thus, V-SVZ and SGZ NSCs share a common transcriptional state throughout their lifespans and transition into and out of dormancy via similar trajectories.

## Significance Statement

This work furthers our understanding of the molecular similarities and differences between the two major populations of adult neural stems [neural stem cell (NSC)] in the mammalian brain: ventricular-subventricular zone (V-SVZ) NSCs and subgranular zone (SGZ) NSCs. We have analyzed high throughput single-cell RNA-sequencing (scRNA-Seq) data for these two NSC populations from embryogenesis through to adulthood and show that while not identical, both populations exhibit a conserved forebrain NSC signature and are transcriptionally similar throughout their lifespans despite the different types of neurons they generate. Moreover, we show that both populations progress from active embryonic precursors to postnatal dormant NSCs along a similar timeframe, and that in both cases reactivation involves a transition back to a development-like state.

## Introduction

Genesis of adult neural stem cells (NSCs) from embryonic neural precursors is an essential developmental process that ensures the continued production of newborn neurons and glia throughout postnatal and adult life. The adult murine brain contains at least two well-characterized NSC populations, one that resides in the ventricular-subventricular zone (V-SVZ) of the lateral ventricles and a second that resides in the subgranular zone (SGZ) of the hippocampal dentate gyrus. These V-SVZ and SGZ NSCs are functionally distinct and generate different types of neurons and glia; V-SVZ NSCs produce inhibitory olfactory bulb (OB) interneurons and oligodendrocytes (Lois and Alvarez-Buylla, 1994; Lois et al., 1996; Menn et al., 2006), whereas SGZ NSCs produce excitatory granule neurons and astrocytes (Brandt et al., 2003; Bonaguidi et al., 2011). However, despite this differential cell genesis, these two NSC populations originate from embryonic neural precursors that reside in adjacent regions of the forebrain lateral ventricles (Young et al., 2007; Fuentealba et al., 2015; Berg et al., 2019). V-SVZ NSCs derive from embryonic cortical and ganglionic eminence (GE) radial glial precursor cells (RPs), whereas SGZ NSCs derive from a subpopulation of embryonic hippocampal precursors in the dentate neuroepithelium (Berg et al., 2018).

How similar are SGZ and V-SVZ NSCs, and what accounts for their functional differences? One idea is that these two NSC types are predetermined by morphogenic cues during early development. For example, lineage tracing and fate mapping studies suggested that V-SVZ NSCs originate from a subset of RPs that are set aside during mid-to-late embryogenesis (Fuentealba, et al., 2015; Furutachi et al., 2015), coincident with acquisition of a slowly-proliferating/quiescent-like cell cycle state (Fuentealba et al., 2015; Furutachi et al., 2015; Yuzwa et al., 2017). However, more recent studies suggest that these embryonic RPs transition to a dormant adult V-SVZ NSC transcriptional state over a relatively lengthy period of time that extends from late embryogenesis to the third postnatal week (Borrett et al., 2020). Similar findings have recently been reported for SGZ NSCs. Lineage tracing and clonal analysis showed that SGZ precursors enter a quiescent-like state by early postnatal development (Berg et al., 2019) and single-cell transcriptional profiling showed that newborn and three-week-old SGZ NSCs are transcriptionally distinct (Hochgerner et al., 2018). Thus, adult V-SVZ and SGZ NSC populations both apparently acquire their adult dormant states at similar times between birth and the third postnatal week. However, despite this similarity, we do not yet know whether the transition to dormancy is similar for these two adult NSC populations, and/or to what extent they or their embryonic precursor parents resemble each other.

Here, we have addressed these questions by computationally comparing the transcriptional profiles of V-SVZ and SGZ-derived NSCs from embryogenesis to adulthood. These analyses indicate that although these two NSC populations produce distinct cellular progeny, they share significant transcriptional commonalities from embryogenesis through to adulthood. Moreover, both populations display a similar developmental transition to dormancy, and reacquire their embryonic states when activated to generate adult-born progeny. These findings therefore support a model where forebrain NSCs are substantively similar at the transcriptional level, and where genesis of their distinct adult-born progeny may at least in part be determined by their adult niche environments.

## Materials and Methods

### Tissue preparation, fluorescence *in situ* hybridization (FISH), and immunostaining

Under RNase-free conditions, brains were harvested from postnatal day (P)5 CD1 mice, fixed in 4% paraformaldehyde (PFA) for 24 h at 4°C, washed in HBSS, transferred to 30% sucrose for 48 h at 4°C, embedded in optimum cutting temperature (O.C.T.) mounting medium (Tissue-Tek) and stored at −80°C. Frozen embedded brains were sectioned coronally at 14-μm thickness and stored at −80°C.

RNA was detected using the RNAscope Multiplex Fluorescent Assay kit (Advanced Cell Diagnostics) under RNase-free conditions. Sections were dried for 15 min at 37°C, washed in PBS for 5 min, then washed in 50%, 70%, and 100% ethanol sequentially for 5, 5, and 2 × 5 min, respectively. After air drying at room temperature, sections were permeabilized using a 1:30 dilution of the RNA-scope Pretreatment-4 protease solution (Advanced Cell Diagnostics) for 10 min at 37°C, washed, and maintained in PBS until probe addition. RNA probes were preheated at 40°C for 10 min and added to the sections and incubated for 2 h at 40°C. Probes were used to target Ptprz1 (catalog #460991, NM_001081306.1), Ttyh1 (504051-C3, NM_001001454.4), Rgs5 (catalog #430181, NM_009063.3), Aldoc (429531-C3, NM_009657.3), and Mt3 (catalog #504061, NM_013603.2) mRNAs. Following probe incubation, sections were washed as recommended by the manufacturer and incubated with RNAscope AMP1 solution for 30 min at 40°C, washed, incubated with RNAscope AMP2 solution for 15 min at 40°C, washed, incubated with RNAscope AMP3 solution for 30 min at 40°C, washed, incubated with RNAscope AMP4 solution for 15 min at 40°C, and washed. For concomitant immunostaining, sections were washed once with PBS, incubated in 5% BSA blocking buffer at room temperature for 1 h, and incubated in primary antibody solution (goat anti-Sox2 diluted 1:1000 in 2.5% BSA; R&D Systems) overnight at 4°C in a humidified chamber. Following primary antibody incubation, sections were washed three times with PBS and incubated in fluorescently labeled secondary antibody solution (diluted 1:1000 in PBS; Invitrogen) for 1 h at room temperature. Sections were then washed three times with PBS, incubated with 0.5 mg/ml Hoechst 33258 for 5 min at room temperature, washed three times with PBS, and mounted on glass slides using Aqua-Poly/Mount (Polysciences).

### Imaging and microscopy

Images of FISH with immunostaining were collected using a Quorom spinning disk confocal microscope system. Z stacks of confocal images were taken with an optical slice thickness of 0.3 μm, and projected z-stacked images are shown.

### Single-cell RNA-sequencing (scRNA-Seq) data analysis pipeline

**Figure 1.**
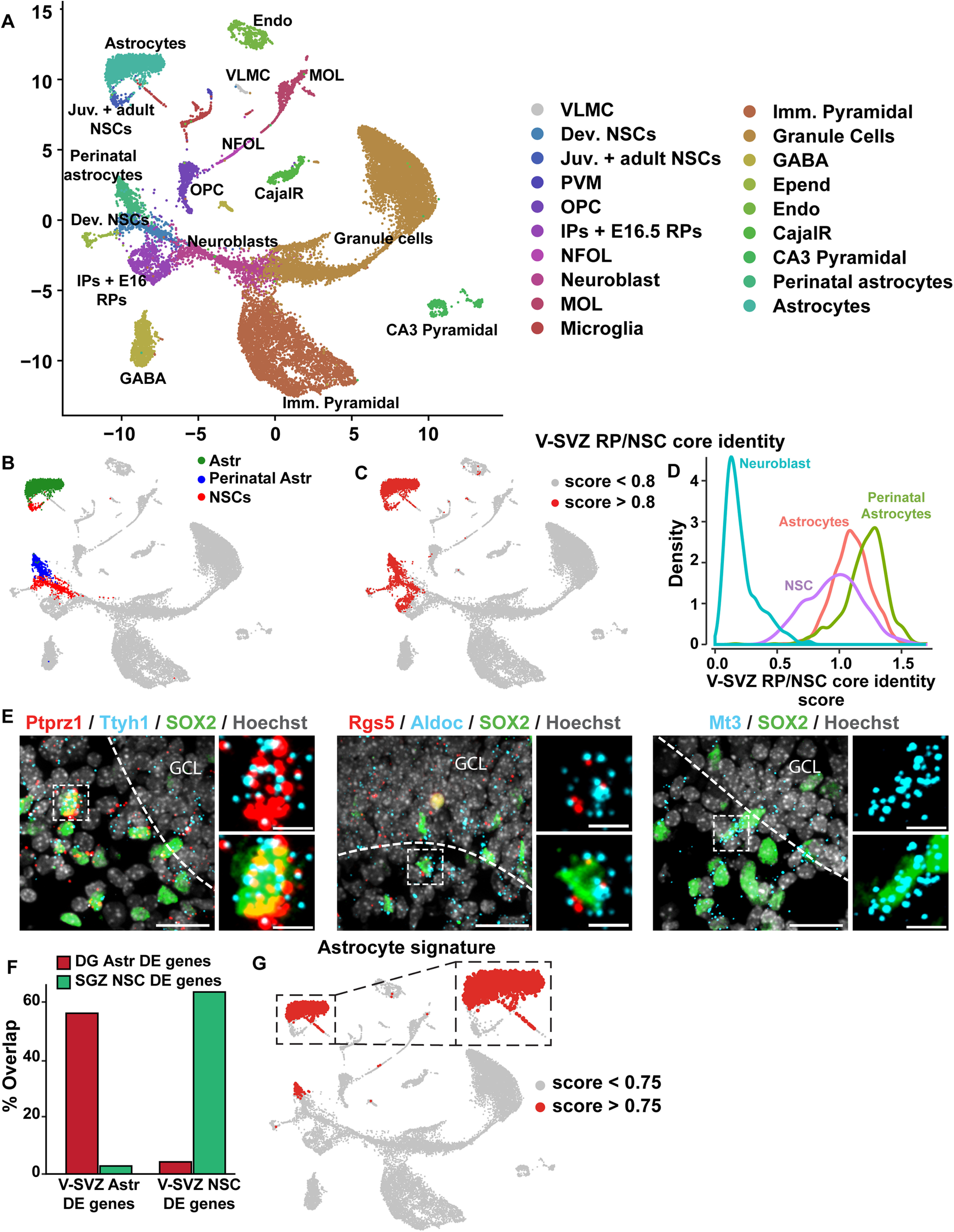
Analysis of single-cell transcriptomes of murine dentate gyrus cells from E16.5 to P132. ***A***, UMAP visualization of dentate gyrus cell transcriptomes from ages E16.5 to P132, colored by cell type using cell annotations described by Hochgerner et al. (2018). Annotations of cell types are shown on the right. VLMC: vascular and leptomeningeal cell; Dev. NSCs: developmental NSCs (E16.5, P0, P5); Juv. + adult NSCs: juvenile and adult NSCs (P18–P132); PVM: perivascular macrophage; OPC: oligodendrocyte precursor cell; IPs: intermediate progenitors (E16.5–P132); RP: radial precursors; NFOL: newly formed oligodendrocytes; MOL: mature oligodendrocytes; Imm. Pyramidal: immature pyramidal cells; GCs: granule cells; GABA: GABAergic neurons; CA3 Pyramidal: pyramidal cells of the hippocampal cornu Ammonis3. Data are not batch-corrected. ***B***, UMAP visualization as shown in ***A*** with the NSCs and astrocytes overlaid in different colors. NSCs at all ages (E16.5–P132) are highlighted in red, perinatal astrocytes (Astr; P0, P5) in blue and juvenile/adult astrocytes (Astr; P18–P132) in green. ***C***, UMAP as shown in ***A*** overlaid with gene expression scores for a previously defined core identity for embryonic cortical RPs and V-SVZ NSCs (V-SVZ RP/NSC core identity; Yuzwa et al., 2017; Borrett et al., 2020). Red denotes cells with scores >0.8. ***D***, Density plot showing the distribution of gene expression signature scores of the V-SVZ RP/NSC core identity as in ***C*** in distinct dentate gyrus populations. SGZ NSCs, perinatal astrocytes (P0, P5), juvenile/adult astrocytes (P18–P132) and neuroblasts are shown and are color coded. ***E***, Confocal z-stack images of coronal sections through the P5 dentate gyrus analyzed by FISH with probes for *Ptprz1*, *Ttyh1*, *Rgs5*, *Aldoc*, and *Mt3* mRNAs (red or blue dots), combined with immunostaining for Sox2 (green) and counterstained with Hoechst (gray). Hatched white lines outline the border between the SGZ and the granule cell layer (GCL) and hatched boxes denote single labeled cells that are shown at higher magnification on the right. Scale bars represent 20 μm in the lower magnification images and 5 μm in the magnified images. ***F***, Bar graph showing the proportion of differentially expressed genes between V-SVZ astrocytes (V-SVZ Astr) and V-SVZ NSCs that are also differentially expressed between SGZ NSCs and dentate gyrus astrocytes (DG Astr); 64% of genes enriched in V-SVZ NSCs relative to V-SVZ astrocytes (V-SVZ NSC DE genes) were also enriched in SGZ NSCs relative to dentate gyrus astrocytes, while 56% of genes enriched in V-SVZ astrocytes relative to V-SVZ NSCs (V-SVZ Astr DE genes) were also enriched in dentate gyrus astrocytes relative to SGZ NSCs. ***G***, UMAP visualization as in ***A*** overlaid with gene expression scores for a 26 gene signature specific to astrocytes relative to NSCs in the V-SVZ and SGZ. These 26 genes are highlighted with asterisks in Table 2. The region shown in the hatched box includes juvenile/adult astrocytes and NSCs as identified in ***B*** and is shown at a larger size to the right. Red denotes cells with scores >0.75.

**Figure 2.**
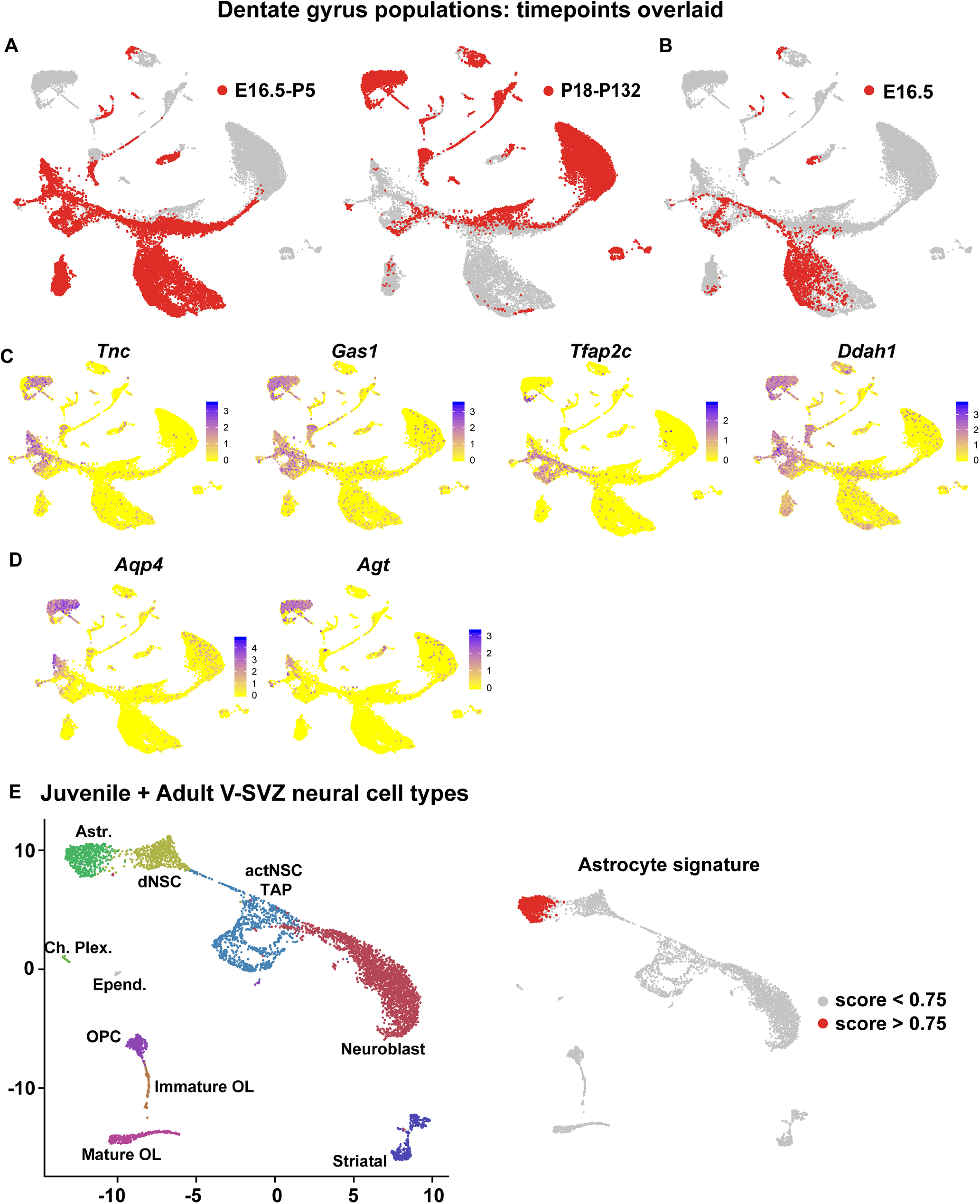
Molecular distinctions between NSCs and astrocytes are conserved in the V-SVZ and SGZ. ***A***, ***B***, UMAP visualizations of dentate gyrus cells from E16.5 to adulthood as in Figure 1*A*, overlaid to show cells from different age groups (red), including all developing cells from E16.5 to P5 (***A***, left panel), juvenile/adult cells from P18 to P132 (***A***, right panel), and E16.5 alone (***B***). Data are not batch-corrected. ***C***, UMAP visualizations as shown in Figure 1*A*, overlaid for expression of four V-SVZ RP/NSC core identity genes. Cells are color-coded for levels of expression as per the adjacent color keys. ***D***, UMAP visualizations as in Figure 1*A*, overlaid for expression of two astrocyte-enriched mRNA from the astrocyte gene signature, *Aqp4* and *Agt.* Cells are color-coded for levels of expression as per the adjacent color keys. ***E***, UMAP visualization of transcriptomes of juvenile/adult (P20, P34, P61) neural V-SVZ cells from Borrett et al. (2020), annotated for cell types. Astrocytes: Astr.; dNSC: dormant NSCs; actNSC: activated NSC; transit amplifying cells: TAP; choroid plexus: Ch. Plex.; ependymal cells: Epend.; oligodendrocyte progenitor cells: OPC; oligodendrocyte: OL; striatal neurons; Striatal. UMAP on the right is overlaid for the 26 gene signature specific to niche astrocytes, where red denotes cells with scores >0.75. Data are not batch-corrected.

Hippocampal dentate gyrus scRNA-Seq data described in Hochgerner et al. (2018) was downloaded from GSE95753. 10× Genomics scRNA-Seq dataset in Hochgerner et al. (2018), termed “Dataset C” and consisting of 24,185 cells (GSE104323), was used for all described analyses below. Dataset count matrix was imported into Seurat version 3.1.1 and was normalized using Seurat’s library size normalization method. Genes detected in fewer than three cells were removed from the dataset. PCA was performed using highly variable genes in the data. The Seurat function RunUMAP was used to generate two-dimensional UMAP projections using the top principal components detected in the dataset. UMAP visualization of all dentate gyrus cell types was subsequently overlaid with specific cell types annotated by Hochgerner et al. (2018) as shown in Figure 1*A* to ensure reproducibility of data analysis. Annotations by Hochgerner et al. (2018) can be found at GSE104323. The P20, P34, P61 merged V-SVZ neural cell dataset described in Borrett et al. (2020) was also run through this computational pipeline as shown in Figure 2*E* to more consistently compare the V-SVZ cell types with the dentate gyrus populations.

**Figure 3.**
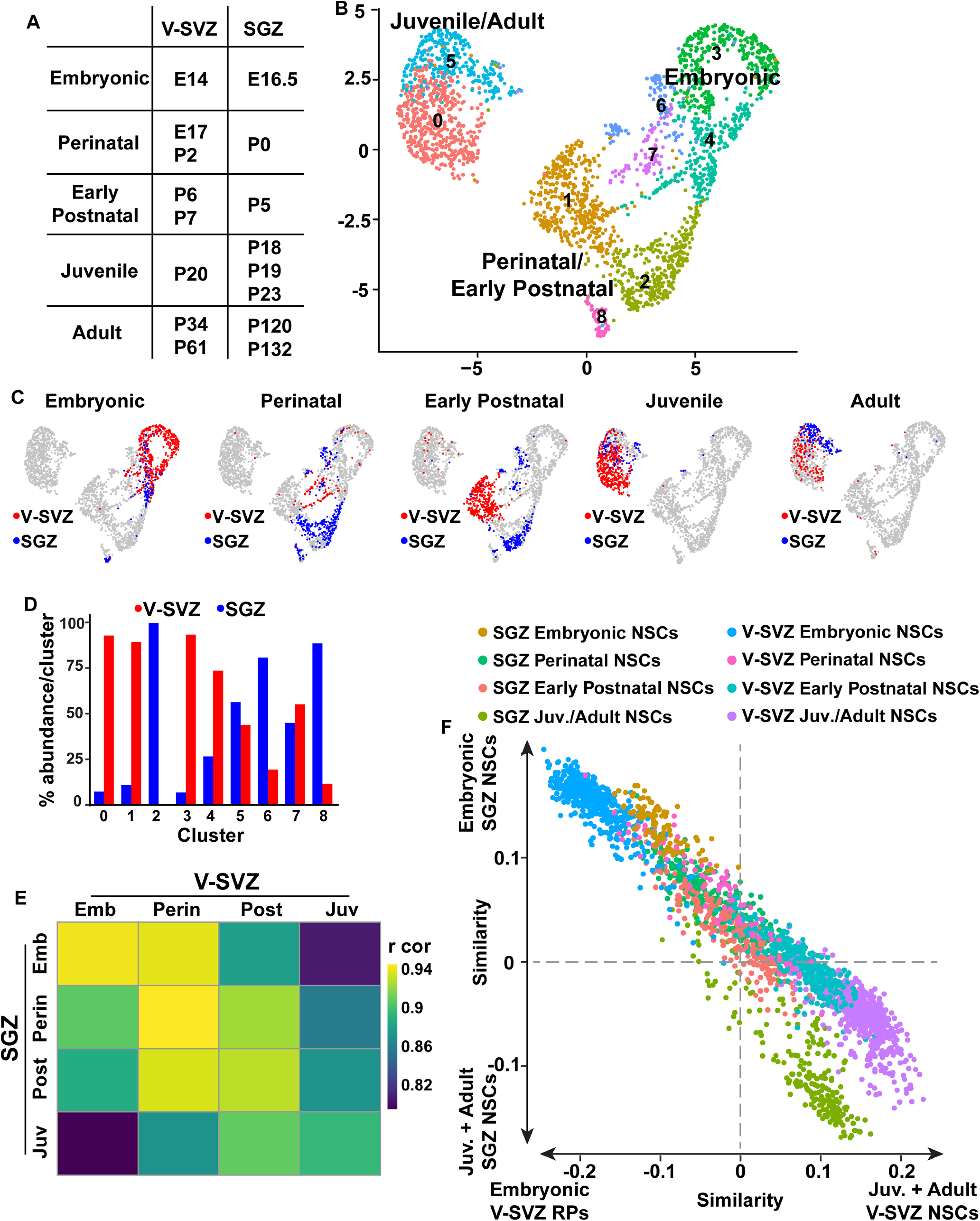
Comparison of V-SVZ and SGZ RP/NSCs from embryogenesis to adulthood. ***A***, Table illustrating the assignment of age-related categories to V-SVZ and SGZ derived RP/NSCs at various timepoints from E14 to P132. ***B***, Batch-corrected UMAP visualization of merged V-SVZ NSCs (*n* = 1594) and SGZ NSCs (*n* = 885) from all ages depicted in ***A***. Cells were grouped into color coded and numbered clusters based on gene expression profiles. ***C***, UMAPs as in ***B*** showing V-SVZ and SGZ NSCs from the different age groups as defined in ***A***. V-SVZ NSCs are shown in red and SGZ NSCs are shown in blue. ***D***, Bar graph showing the percentages of V-SVZ and SGZ transcriptomes in each of the clusters shown in ***B***. V-SVZ proportions are shown in red and SGZ proportions are shown in blue. ***E***, Correlation heatmap showing Pearson correlation coefficients between V-SVZ and SGZ NSC average gene expression profiles from the different age groups shown in ***A***. Gene expression values are not batch-corrected. Correlation coefficients are color coded as per the adjacent color key. Emb: embryonic; Perin: perinatal; Post: early postnatal; Juv: juvenile. ***F***, Scatterplot showing single**-**cell correlation analysis of transcriptomes from embryonic, perinatal, early postnatal, juvenile and adult V-SVZ and SGZ RP/NSCs (as defined in ***A***), where the individual transcriptomes were each correlated with the averaged gene expression for E14 V-SVZ RPs versus juvenile/adult V-SVZ dormant NSCs (P20, P34, P61; *x*-axis) and with the averaged gene expression for E16.5 SGZ RPs versus juvenile/adult SGZ NSCs (P18, P19, P23, P120, P132; *y*-axis). Gene expression values are not batch-corrected. Cells are color coded for their dataset and age of origin. Juvenile and adult SGZ NSCs are represented in the same color. Juvenile and adult V-SVZ NSCs are represented by the same color.

To generate the SGZ NSCs and V-SVZ NSCs merged dataset shown in Figure 3*B*, SGZ NSCs (also known as radial glial like cells; RGL) from all timepoints (885 total cells), annotated by Hochgerner et al. (2018) were extracted from the complete dentate gyrus dataset, and cell transcriptomes were merged with V-SVZ NSC transcriptomes described in Borrett et al. (2020) and subsequently run through a batch corrected version of the pipeline described above (methods described below) resulting in 2479 total forebrain NSCs. Cell cycle regression was performed on the same dataset (method described below). The same top principal components used to perform UMAP dimensionality reduction, were subsequently used to iteratively carry out SNN-Cliq-inspired clustering (FindClusters function in Seurat). Clusters were assigned at a resolution of 0.4 (nine clusters identified without cell cycle regression and eight clusters identified with cell cycle regression, presumably because of reduced cell-cycle related clustering). To generate the E14 RP and embryonic day (E)16.5 dentate neuroepithelium RP merged dataset shown in Figure 5*A*, all E14 RP (cortical and GE-derived) and E16.5 dentate neuroepithelium RP raw transcriptomes were extracted and run through the same batch corrected pipeline. To generate the P6/P7 V-SVZ NSC and P5 SGZ NSC merged dataset shown in Figure 5*E*, all P6/P7 RP (cortical and GE-derived) and P5 SGZ NSC raw transcriptomes were extracted and run through the same batch corrected pipeline. Juvenile and adult transit-amplifying precursors (TAPs)/intermediate progenitors (IPs) of both V-SVZ and SGZ origin were combined to the merged NSC dataset shown in Figure 3*B* and were subsequently run through the batch corrected pipeline. SGZ IPs included all IPs annotated by Hochgerner et al. (2018) from P18 to P132 and V-SVZ TAPs included all nonproliferative TAPs at P20,P34,P61 identified in Borrett et al. (2020). This population of TAPs included a small number of activated NSCs at these ages as described in Borrett et al. (2020). Note that for all dataset merging, the union of all detected genes from each dataset was always used. tSNE gene overlays were generated using the Seurat FeaturePlot function, violin plots were generated using the Seurat VlnPlot function, heatmaps were generated using the Seurat DoHeatmap function (using scaled expression values).

### Batch correction of merged V-SVZ and SGZ datasets

V-SVZ and SGZ cell transcriptomes were corrected for batch differences using the R program Harmony (version 1.0; Korsunsky et al., 2019). Cells were categorized into two distinct batches based on their site of origin: all V-SVZ cells were considered batch 1 and all SGZ cells were considered batch 2. Following PCA, a single iteration of Harmony-mediated correction was performed on the normalized cell transcriptomes based on the above batch categorization using the RunHarmony function. Following Harmony batch correction, both UMAP dimensionality reduction and SNN-Cliq inspired clustering were performed using the obtained “harmonized” principal component space as opposed to the original uncorrected principal component space. This same protocol was conducted for all datasets on which batch correction was performed.

### Trajectory inference and pseudotime ordering

Single-cell pseudotime trajectories were constructed as described in Borrett et al. (2020) using a modified version of the dpFeature method in Monocle v2 (Trapnell et al., 2014) as previously published (Borrett et al., 2020; Storer et al., 2020). Briefly, cell barcodes from desired datasets were extracted from the raw digital gene expression matrices and merged before normalization using Monocle’s size factor normalization method. PCA was performed using the same highly variable genes that were obtained from our custom-built pipeline as described above and the cells were projected into two-dimensional space using the tSNE algorithm. Cells were subsequently assigned into distinct clusters using Monocle’s density peak clustering algorithm. A set of ordering genes was obtained by testing each gene for differential expression between the clusters in the dataset and selecting the top 1000 significantly differentially expressed genes. Expression profiles were reduced to two dimensions using the DDRTree algorithm included in Monocle 2 and cells were ordered using these genes to obtain a trajectory. Cell cycle regression was performed as described below.

### Cell cycle regression analysis

Cell cycle regression was conducted using the same method described in Borrett et al. (2020) by removing all cell cycle related genes from the highly variable genes used to perform PCA. All downstream steps were performed as previously described. In order to carry out cell cycle regression on the trajectory inference analysis performed using Monocle, the same list of cell cycle related genes was removed from the top 1000 differentially expressed genes used to order the cells along the inferred trajectory. In order to obtain a list of cell cycle related genes, we took the enriched genes from all G1, S, and G2/M marker gene pairs used by the Cyclone method (Scialdone et al., 2015) to assign cell cycle phase that were detected in our scRNA-Seq dataset. These genes were subsequently combined with an additional list of S phase related and G2/M phase related genes described in Kowalczyk et al. (2015). Together, this resulted in a total of 678 cell cycle related genes that were used to perform cell cycle regression.

### Gene set enrichment analysis (GSEA)

GSEA on the SGZ NSCs was performed using the same computational method described in Borrett et al. (2020). Gene correlation with time was performed by converting developmental day for each cell to an integer value, with birth at zero, then calculating Spearman rank correlation of normalized gene expression for each gene with time. GSEA was performed on the correlation coefficients as per the protocol in Reimand et al. (2019), using the quiescence gene set (Cheung and Rando, 2013) and gene sets defined here: http://download.baderlab.org/EM_Genesets/January_01_2020/Mouse/symbol/Mouse_GOBP_AllPathways_no_GO_iea_January_01_2020_symbol.gmt. GSEA calculations were performed in R using the fast GSEA (fgsea) algorithm. Large gene set databases contain redundancy that makes interpretation difficult, so before reporting enriched gene sets, the results were collapsed into a non-redundant set (minimizing overlapping genes per set) using a Bayesian network construction approach (Korotkevich et al., 2021).

### Upregulation of quiescence genes over time

Upregulation of quiescence genes over time shown in Figure 6*E* was performed as described in Borrett et al. (2020). Gene correlation with time was performed by converting developmental day for each cell to an integer value, with birth at zero, then calculating Spearman rank correlation of normalized gene expression for each gene with time (same method as was done in the GSEA). Quiescence genes (defined in Cheung and Rando, 2013) were determined to be more correlated with time by comparing Spearman rank correlation coefficients versus all other detected genes using the Wilcoxon rank-sum test. Significance values are given in the figure legend and Results.

### Pearson correlation analysis

Average Pearson correlation analysis was conducted by averaging the expression of each gene in a given cluster or cell type (i.e., population at a given age) and performing Pearson correlation using the cor.test function in R. Correlation coefficients between different populations were subsequently displayed as heatmaps using the pheatmap package in R. The single-cell Pearson correlation analysis depicted in Figures 3*F*, 7*A* was conducted as described in previous studies (Borrett et al., 2020; Storer et al., 2020; Toma et al., 2020). Average transcriptomes were calculated for juvenile and adult V-SVZ dNSCs, E14 total cortical and GE RPs, juvenile and adult SGZ NSCs, and E16.5 dentate gyrus RPs by averaging the expression of the union of all detected genes in each of the four cell populations. Each cell depicted on the plot was subsequently correlated to each of the four average transcriptomes using Pearson correlation (cor function in R). *X*-coordinates represent the difference between the correlation of a cell with the juvenile and adult V-SVZ dNSC average transcriptome and the correlation of the same cell with the E14 total RP average transcriptome. Y-coordinates represent the difference between the correlation of a cell with the E16.5 dentate gyrus RP average transcriptome and the correlation of the same cell with the juvenile and adult SGZ NSC average transcriptome.

### Differential gene expression statistical analysis

Differential expression (DE) was performed as described in Borrett et al. (2020). Statistics used to test differential gene expression in the scRNA-Seq data were performed using the Seurat FindMarkers function using a Wilcox test (Seurat version 3.1.1). An adjusted *p* value (Family-Wise Error Rate; FWER) smaller than 0.05 was considered statistically significant (Bonferroni correction).

### NSC versus astrocyte molecular comparison

Differential gene expression analysis was performed between all SGZ NSCs at all ages with all dentate gyrus niche astrocytes at all ages as described above. These genes were compared with the differentially expressed genes between V-SVZ NSCs and V-SVZ niche astrocytes at P20, P34, and P61 (analysis previously performed in Borrett et al., 2020). The overlap of astrocyte-enriched genes and NSC-enriched genes in both regions was subsequently determined. The overlapping proportions are shown in Figure 1*F*. Of the overlapping astrocyte-enriched genes, 26 genes that exhibited the most specific expression to astrocytes were selected as a means to define a molecular signature that labels forebrain astrocytes and not forebrain NSCs. These genes included *Aqp4*, *Slc4a4*, *Gjb6*, *Grin2c*, *Abhd3*, *Cxcl14*, S100β, *Fgfr3*, *Cadm2*, *Slc39a12*, *Tril*, *Hapln1*, *Arxes2*, *Gabrg1*, *Car2*, *Pfkp*, *Lcat*, *Hsd11b1*, *Cryab*, *Vegfa*, *Timp4*, *AI464131*, *Omg*, *Syne1*, *Cd38*, and *Agt*.

### Shared adult dormant NSC gene signature analysis

In order to compute the shared adult NSC signature described in Figures 8, 9, differential expression analysis was conducted as described above between embryonic RPs, juvenile/adult dormant NSCs and juvenile/adult TAPs/IPs of both V-SVZ and SGZ origin. Genes upregulated (> 0.5 avg log fold change, adj. *p* value < 0.05; FWER) in adult dormant NSCs relative to both embryonic RPs and adult TAPs/IPs were computed for both V-SVZ and SGZ populations. V-SVZ and SGZ genes identified by this analysis were compared and the overlap of both gene sets were termed the shared adult dormant NSC signature. This consisted of a total of 94 genes as shown in Tables 6, 7.

### Quantification of gene signature

Quantification of gene signatures in cell types was performed as described in Borrett et al. (2020). Gene signature scores were computed by taking the average expression of all detected signature genes in each cell. Gene signature scores for each cell were subsequently overlaid on the tSNE plot to display cells with the highest signature scores. This analysis was conducted for three different gene signatures: (1) a cortical RP core identity signature identified in Yuzwa et al. (2017); (2) the astrocyte gene signature described above; and (3) the shared adult dormant NSC signature described above. Expression cut-offs are provided in the figures and legends. Density plots showing distribution of signature scores were performed using ggplot.

## Results

### A V-SVZ NSC core transcriptional signature is conserved in developing and adult SGZ NSCs

To compare V-SVZ and SGZ NSCs, we used two recently-published single-cell transcriptome datasets, one including forebrain V-SVZ cells from E14 to P61 (Borrett et al., 2020) and a second including dentate gyrus cells from E16.5 to P132 (Hochgerner et al., 2018). Since these datasets were generated using two different protocols, we ensured that they were comparable by analyzing both of them using a slightly modified version of a previously described scRNA-Seq computational pipeline (see Materials and Methods for details; Yuzwa et al., 2017; Carr et al., 2019; Borrett et al., 2020; Storer et al., 2020). This pipeline was originally described in Yuzwa et al. (2017) and incorporates extensive low level data quality analysis and evidence-based parameter selection to visualize and cluster transcriptomes from scRNA-Seq datasets. For the hippocampus, we used this pipeline to analyze the 24,185 dentate gyrus transcriptomes of all ages from Hochgerner et al. (2018; termed Dataset C in Hochgerner et al., 2018; GSE 95 753). Following analysis, we used UMAPs to visualize clustering and were able to identify transcriptome clusters corresponding to both neural and nonneural cell types (Figs. 1*A*, 2*A*), as previously described (Hochgerner et al., 2018). Of particular relevance, we found that neonatal NSCs (P0 and P5) and nonproliferative E16.5 RPs (together labeled developing NSCs) were co-clustered and were distinct from clusters containing the P18 and older NSCs (P18, P19, P23, P120, and P132; labeled adult NSCs). There was also a population of proliferative E16.5 RPs that were co-clustered with P0 and P5 cells that were previously-defined as transit-amplifying IPs (Figs. 1*A*, 2*B*; labeled IPs + E16 RPs). All of the precursor clusters were segregated from two additional distinct clusters containing perinatal astrocytes (P0 and P5) and juvenile/adult astrocytes (P18 and older; labeled Astrocytes; Fig. 1*A*,*B*). This clustering analysis therefore suggests that juvenile and adult SGZ NSCs are very similar to each other but are quite distinct from embryonic and perinatal SGZ NSCs, a finding previously reported in Hochgerner et al. (2018).

To start to ask about potential transcriptional similarities between SGZ and V-SVZ NSCs, we examined 79 genes that were first identified as highly enriched in embryonic cortical RPs relative to all other embryonic cortex cell types (Yuzwa et al., 2017) and then were shown to also be enriched in postnatal V-SVZ NSCs (Borrett et al., 2020; see Table 1 for a list of the differentially-expressed genes). We used these 79 genes to compute a single-cell gene expression score and applied this to all of the cells in the dentate gyrus dataset (Fig. 1*C*,*D*). The gene signature was enriched in developing and adult SGZ NSCs, and in all E16.5 hippocampal RPs. To confirm this result, we also analyzed average expression levels for these 79 genes. This analysis showed that 63 of the 79 genes were enriched in nonproliferative SGZ NSCs (cells highlighted in red in Fig. 1*B*; Table 1) relative to the collection of the remaining dentate gyrus cells (as shown in Fig. 1*A*; adjusted *p* value < 0.05; FWER). The genes that were not enriched were *NdeI, Rgcc, Ednrb, Metrn, Kbtbd11, Gm11627, Acadl, Aldhl11, Bcan, Vit, Acss1, Acsbg1, Atp1a2, Clu, Pnp, and* *Rcn3*.

**Table 1:** Expression of V-SVZ RP/NSC core identity genes in hippocampal SGZ NSCs and astrocytes (related to Fig. 1)

Core genes	SGZ NSC abundance (%)	SGZ Astr abundance (%)
Acaa2	21.6	17.3
Aldoc	85.1	98.7
Apoe	90.8	99.9
Asrgl1	42.9	63.4
Ccdc80	49.0	19.0
Cd63	65.6	64.2
Ckb	87.7	94.6
Cyr61	34.5	12.2
Dbi	99.2	92.8
Ddah1	72.4	59.3
Efhd2	40.7	36.4
Fabp7	94.7	75.3
Fgfbp3	28.9	16.7
Gas1	66.9	33.9
Gng12	51.2	53.6
Gpx8	30.4	12.1
Gsta4	29.3	11.6
Hes1	45.3	28.2
Hes5	63.6	47.9
Hopx	58.0	42.1
Id1	34.0	26.8
Id3	54.6	62.0
Id4	36.4	59.1
Lfng	32.2	26.5
Magt1	24.5	25.2
Mdk	72.8	43.5
Mfge8	78.8	82.9
Mlc1	51.2	79.4
Mt1	94.5	98.3
Mt2	85.6	93.3
Mt3	94.0	99.8
Myo10	27.2	38.8
Nek6	28.4	9.2
Nes	15.4	3.5
Nr2e1	29.5	20.4
Nrarp	43.3	34.8
Oat	37.6	45.2
Pax6	55.0	32.6
Pdpn	37.5	34.0
Pea15a	70.8	62.3
Phgdh	66.8	53.0
Pon2	39.3	45.6
Psat1	57.3	46.0
Ptprz1	93.1	95.6
Rcn1	35.7	13.8
Rhoc	32.0	18.3
Serpinh1	38.8	24.9
Sfrp1	33.9	6.2
Slc1a3	97.5	99.6
Slc9a3r1	47.3	54.2
Sox2	59.8	65.3
Sox21	20.7	29.2
Sox9	78.2	77.1
Sparc	55.5	22.8
Tead2	31.3	7.0
Tfap2c	38.6	0.7
Tgfb2	38.6	21.6
Tnc	50.5	29.0
Ttyh1	75.0	96.5
Vcam1	33.2	48.3
Veph1	24.6	1.7
Vim	66.4	18.0
Zfp36l1	70.8	45.1

Shown are 63 of the 79 embryonic cortical signature genes defined in Yuzwa et al. (2017) that are not cell cycle associated and are significantly enriched in SGZ NSCs (cells highlighted in red in Fig. 1*B*) relative to all other combined cell types in the dentate gyrus from embryogenesis through to adulthood (E16.5–P132; adjusted *p* value, FWER < 0.05). The relative proportions of SGZ NSCs (red cells in Fig. 1*B*) and all SGZ astrocytes (green and blue cells in Fig. 1*B*) that detectably express these mRNAs are also shown.

These data suggest that a similar core gene signature is enriched in V-SVZ and SGZ precursors from embryogenesis through to adulthood. We validated expression of a subset of these genes in SGZ NSCs by performing FISH for *Ptprz1*, *Ttyh1*, *Aldoc*, and *Mt3* on the neonatal P5 dentate gyrus. To identify NPCs, we combined the FISH with immunostaining for the precursor protein Sox2. As predicted by the scRNA-Seq analysis, there were Sox2-positive cells within the developing SGZ that co-expressed these different mRNAs (Fig. 1*E*).

### Defining a gene signature that distinguishes niche astrocytes from NSCs

One limitation of this analysis is that the V-SVZ RP/NSC gene signature, as well as many of the individual genes, were also enriched in SGZ niche astrocytes (Fig. 1*C,D*; Table 1), as previously observed in the V-SVZ (Borrett et al., 2020). For example, *Tnc*, *Gas1*, and *Ddah1* mRNAs were enriched in both astrocytes and NSCs, although some mRNAs, such as *Tfap2c*, *Vimentin* (*Vim*), and *Nestin* (*Nes*) were more specific to the NSCs (Fig. 2*C*; Table 1). We therefore asked whether we could identify genes that more definitively distinguished NSCs from niche astrocytes in the SGZ by focusing on a gene set recently shown to be differentially expressed in these two cell types in the P20–P61 V-SVZ. This gene set included 537 mRNAs that were significantly higher in their expression in V-SVZ astrocytes versus NSCs, and 498 genes that were significantly lower (Borrett et al., 2020). Analysis of these same genes in the dentate gyrus dataset (Fig. 1*F*) showed that 64% of the genes that were expressed more highly in V-SVZ NSCs were also expressed at higher levels in SGZ NSCs than in SGZ astrocytes, while 56% of genes that were higher in V-SVZ astrocytes were also higher in SGZ astrocytes (Fig. 1*F*; Table 2).

**Table 2:** Genes that are differentially expressed between NSCs and astrocytes in both the V-SVZ and SGZ (related to Fig. 1, 2)

Astr-enriched genes in V-SVZ + SGZ	NSC-enriched genes in V-SVZ + SGZ
Gpr37l1	Dbi
Sparcl1	Sfrp1
Cxcl14*	Rpl41
Htra1	Rplp0
Bcan	Rps27a
Id2	Rpl18a
Aqp4*	Rps27
Tril*	Rpl35a
Ntsr2	Rps19
Atp1b2	Rpl13a
Timp4*	Rpl9
Car2*	Rpl3
Atp1a2	Rps14
Eno1	Eef1a1
Kcnk1	Rpl13
S100b*	Rplp1
Dbx2	Ptma
Cldn10	Rps4x
Btbd17	Rpl10
Aplp1	Marcksl1
Slc39a12*	Rpl23a
Msmo1	Rps24
Gja1	Rpl17
Slc7a10	Rps5
Lsamp	Rpl14
Pla2g7	Vim
Fjx1	Rps9
Gria2	Rps23
Plpp3	Rps15a
Abhd3*	Rps18
F3	Rpl37
Gpm6a	Rpl11
Dclk1	Rpl27a
Clu	Rps16
Gjb6*	Rps8
Slc4a4*	Rpl26
Tmem100	Rpl37a
Omg*	Rps13
Ntm	Rps10
Eva1a	Gnas
Grina	Rps20
Scg3	Rpl32
Arxes2*	Rplp2
S1pr1	Rpl8
Apoe	Rps2
Smpdl3a	Rpl34
Camk2n1	Rpl38
mt-Co3	Rps21
Acsbg1	Rps25
Agpat5	Rpl23
Acsl6	Rps12
Gpc5	Riiad1
Hacd2	Rps3a1
Cadm1	Rpl7
Fgfr3*	Sparc
Aldoc	Rpl10a
Hapln1*	Rps6
Mfge8	Rpl22l1
Hbegf	Rps15
Tuba4a	Rps28
Hsd11b1*	H2afv
Grin2c*	Rps7
Tmem176a	Rps11
Grm3	Rtn1
Chst1	Fau
Slc38a3	Rpl21
Tspan7	Rpl31
Macf.1	Rpl39
Sepp1	Ftl1
Lcat*	Rps17
Clmn	Tmsb4x
Vegfa*	Hmgb1
AI464131*	Rpl30
Slc6a1	Rpsa
Pfkp*	Hsp90aa1
Paqr7	Ccnd2
Eps8	Rpl6
Slc9a3r1	Rps3
Tagln3	Ascl1
Fermt2	Tpt1
mt-Nd1	Rpl36a
Oaf	Fxyd6
Vcam1	Rpl15
Tlcd1	Rpl36
Tmem176b	Fabp7
mt-Atp6	Rpl19
mt-Cytb	Rpl4
mt-Nd2	Bex4
Cryab*	Hmgn1
Serinc1	Cd9
Cd81	Rpl18
Phyhipl	Rpl24
Ptprz1	Pebp1
Ppp1r3g	Psph
Syne1*	Rps26
Cd38*	Ypel3
Mertk	Cnbp
Appl2	Rpl12
Mt1	Rpl22
Mfsd2a	Swi5
Ank2	Zbtb20
Fam20a	Ybx1
Tprkb	Sptssa
Sept7.	Eef2
Pcdh7	Tox3
Scrg1	Slc38a1
Tmed5	Rpl35
Ccdc88a	Naca
Ugp2	Ywhae
mt-Nd4	Plagl1
Cadm2*	Rpl29
mt-Co2	Sept15.
Ptn	Smim11
Mt2	Arl4c
Pmm1	Rpl5
Il18	Fbln2
mt-Co1	Bex2
2900052N01Rik	H3f3a
Apln	Rpl27
Luzp2	Mif
Slc6a11	Maged1
Slco1c1	Marcks
Rgcc	Mrfap1
Ncan	Snrpg
Slc1a3	Mfap2
Id3	Rpl7a
Acsl3	Snrpd2
Phactr3	Veph1
Serpine2	Tuba1a
P4 ha1	Chchd2
Tmem44	Ppia
Agt*	Tomm7
Enho	Jund
Adora2b	Ubl5
Hacd3	Acot1
Tsc22d4	H1f0
Cdh10	Anapc11
Dhcr7	Btf3
Gabrg1*	Hdgf
Ctsd	Pfdn5
Cystm1	Gnb2l1
Phkg1	Trim2
Slc7a11	Tead2
Usp53	Psip1
Pcdh10	Ifitm2
Arhgap5	Pdlim4
Sec14l2	Ap1s2
Nptn	Rcn1
Thy1	Eif3f
Cmtm5	Rpl28
Atp13a4	Cetn2
Elovl2	Clic1
Rorb	Ndn
Fut9	Nenf
Sat1	Snrpe
Pcdh9	Gabarap
Ttyh1	Dek
mt-Nd4l	Prdx2
Pfkm	Eef1d
Gabrb1	Idh2
Fam21	Stra13
Cpeb4	Sh3bgrl3
Prex1	Atpif1
Pmp22	Srp9
Gatm	Nsg1
Csrp1	Hsbp1
Smpd1	Eef1g
Cyp7b1	Serf1
Pcdh17	Myl9
Tlr3	Fam210b
Metrn	Aif1l
Lgr4	Cox7a2l
Chchd10	Bex1
Slc14a1	Dstn
Rrbp1	Tuba1b
Gpr162	Rps27l
Abcd2	Ap2m1
Gpr37	Stmn3
Slitrk2	Ahsa1
Elovl5	Ptx3
Emc3	Trmt112
Tnik	Hmgn2
Saraf	Eef1b2
Cntfr	Creb5
Aco2	Sf3b2
Ubc	Cfdp1
Chst10	Tspan13
Plcd4	Park7
Hmgcr	Ei24
Tmem229a	Sec61g
Gstm5	Fkbp3
Wscd1	Tmem107
Gpi1	Tbca
Stt3b	Snrpf
Hepacam	Anp32b
Cd47	Atp5e
Ednrb	Nudc
Mdga2	Psmg4
Cyp2j6	Pkig
Akt2	Sumo2
Pgm2	Erh
Nebl	Hcf.c1r1
Olig1	2810459M11Rik
Mfn1	Tmem258
Ddhd1	Pfdn2
Trp53bp2	Maf1
Rapgef3	Bnip3l
Crot	Akr1a1
Adk	Rpa2
Rasa2	Rhcg
Ckb	Kif21a
Rnf13	Oaz1
Slc20a1	Sumo3
Dner	2700094K13Rik
Slc27a1	H2afy
Irak2	St13
mt-Nd3	Cetn3
Osbpl1a	Hbb-bs
Cst3	Hint1
Chst2	Efnb1
Nrcam	Tubb5
Tpp1	Fos
Fgf1	Sfr1
Clptm1	Eif1ax
Tmem189	Nedd8
Capn2	Cdc26
Daam2	Elof1
Ndp	Hsp90ab1
Dmd	Ptov1
Slc1a4	Hnrnpc
Hadhb	Rnaseh2c
Nr1d1	Txn1
Baalc	Rnf187
Psd2	Psme1
Aldh1l1	Ngfrap1
Hist1 h1c	Fam32a
Itga6	Nop10
Cyp2d22	Pbx1
Aldoa	Eif3h
Laptm4b	Gltscr2
Cnp	Tmpo
Kifc3	Efnb3
Pcdh1	Aprt
Dnajb9	Psme2
Asah1	Mettl9
Mfap3l	Hmgb2
Camk2g	Rlbp1
Cpq	Slit2
Tank	Use1
Gpr146	Hsd17b10
Pnkd	Hspe1
Mgll	Pter
Arhgef26	Cnpy2
Aifm3	Hnrnpf
Slc2a1	Btg2
Slc41a1	Ywhaq
Fam213a	Psenen
Igsf11	Bri3
Fgfrl1	Wbp5
Adgrl3	Gsta4
Etv5	Trip6
RP23-4H17.3	Mdk
Fut8	Mrpl52
Jam2	Rac3
Kif1b	Ran
Usp54	Eif3i
Sash1	Tma7
Tmbim1	G3bp1
Vcl	Pax6
Ppp3ca	Npm1
Pon2	Chchd7
Phka1	Fkbp4
Chpt1	Ccdc80
Mir124-2 hg	Mbd3
Abi1	Hnrnpr
Uqcr10	Myl6
Stxbp3	Set
Ppp1r1b	Ranbp1
Prex2	Golim4
mt-Nd5	Gpx8
Acss2	Arl3
Tmx2	Bag2
Pid1	Ntan1
Tcn2	Med28
Tfrc	Ddah2
Dio2	Nhp2l1
Trib2	Stk11
Slc15a2	Gpx1
Itpr2	Tsn
Gm2a	Basp1
Npas3	Msn
Pttg1ip	Cers4
Acap2	Unc119
Insig1	Paip2
Csgalnact1	Srp14
Mcur1	Ift22
Uqcr11	Anapc5
S100a13	Hnrnpa1
Retsat	Cnn3
Tmem47	Sumo1
Adgrg1	Gm8730
	Anp32a
	Tceb2
	Myl12a
	Cacybp
	Emg1
	Ssrp1
	Polr3h
	Nfix
	Puf60
	Ppp1ca
	Rpl23a-ps3
	Romo1
	Cfap20
	Gm17750
	Vgll4

Shown are genes that are differentially expressed (FWER < 0.05) between NSCs and astrocytes in both the dentate gyrus and the V-SVZ. Genes identified as differentially expressed by V-SVZ NSCs versus astrocytes in Borrett et al. (2020) were interrogated for their expression in all SGZ NSCs (red cells in Fig. 1*B*) and all SGZ astrocytes (green and blue cells in Fig. 1*B*) in the combined dentate gyrus dataset. The left column indicates genes significantly enriched in astrocytes in both the V-SVZ and SGZ, and the right column indicates genes significantly enriched in NSCs in both the V-SVZ and SGZ. Of the astrocyte-enriched genes, 26 (indicated with asterisks in the table) were highly enriched relative to NSCs, and were used to define a shared forebrain niche astrocyte signature as shown in Figures 1*G*, 2*E*.

This analysis suggests that the same genes that distinguish NSCs from astrocytes in the V-SVZ distinguish these two cell types in the dentate gyrus. To test this idea, we selected 26 of the genes in this dataset that were most highly enriched in astrocytes versus NSCs in both the V-SVZ and SGZ (Table 2, asterisks), as exemplified by the patterns of expression of *Aqp4* and *Agt* (Fig. 2*D*). A gene signature score computed using these 26 genes was specifically enriched in niche astrocytes relative to all other cells in the dentate gyrus dataset (Fig. 1*G*). This gene signature was similarly enriched in V-SVZ niche astrocytes, as shown by computing a similar signature score for the P20, P34, and P61 V-SVZ transcriptomes (Borrett et al., 2020) that had been put through the same computational pipeline (Fig. 2*E*). Thus, while niche astrocytes share many transcriptional commonalities with SGZ and V-SVZ NSCs, astrocytes and NSCs can be readily distinguished at the transcriptional level.

### V-SVZ and SGZ precursors share transcriptional similarities as they progress from active embryonic to dormant adult NSCs

It was previously reported that the transition from an embryonic to adult V-SVZ NSC reflects a switch from an active to a dormant stem cell state, involving a broad dampening of cell biological processes associated with an active state including cell division, transcription, RNA metabolism and protein translation, processing and trafficking (Borrett et al., 2020). The finding that a V-SVZ RP/NSC gene signature is also enriched in SGZ RP/NSCs suggests that these two populations might be more transcriptionally similar than previously appreciated and thus might share similar transcriptional trajectories to a dormant state. To test this idea further, we extracted all nonproliferative SGZ RP and NSC transcriptomes (the red cells in Fig. 1*B*; 885 total cells) and combined them with the V-SVZ RP/NSC transcriptomes (as identified in Borrett et al., 2020), including P2, P6/7, P20, P34, and P61 dormant NSCs and E14 and E17 cortical and GE-derived RPs. This combined dataset was put through the computational pipeline and included V-SVZ and SGZ precursors of similar developmental stages from embryogenesis to adulthood (shown in Fig. 3*A*).

**Figure 4.**
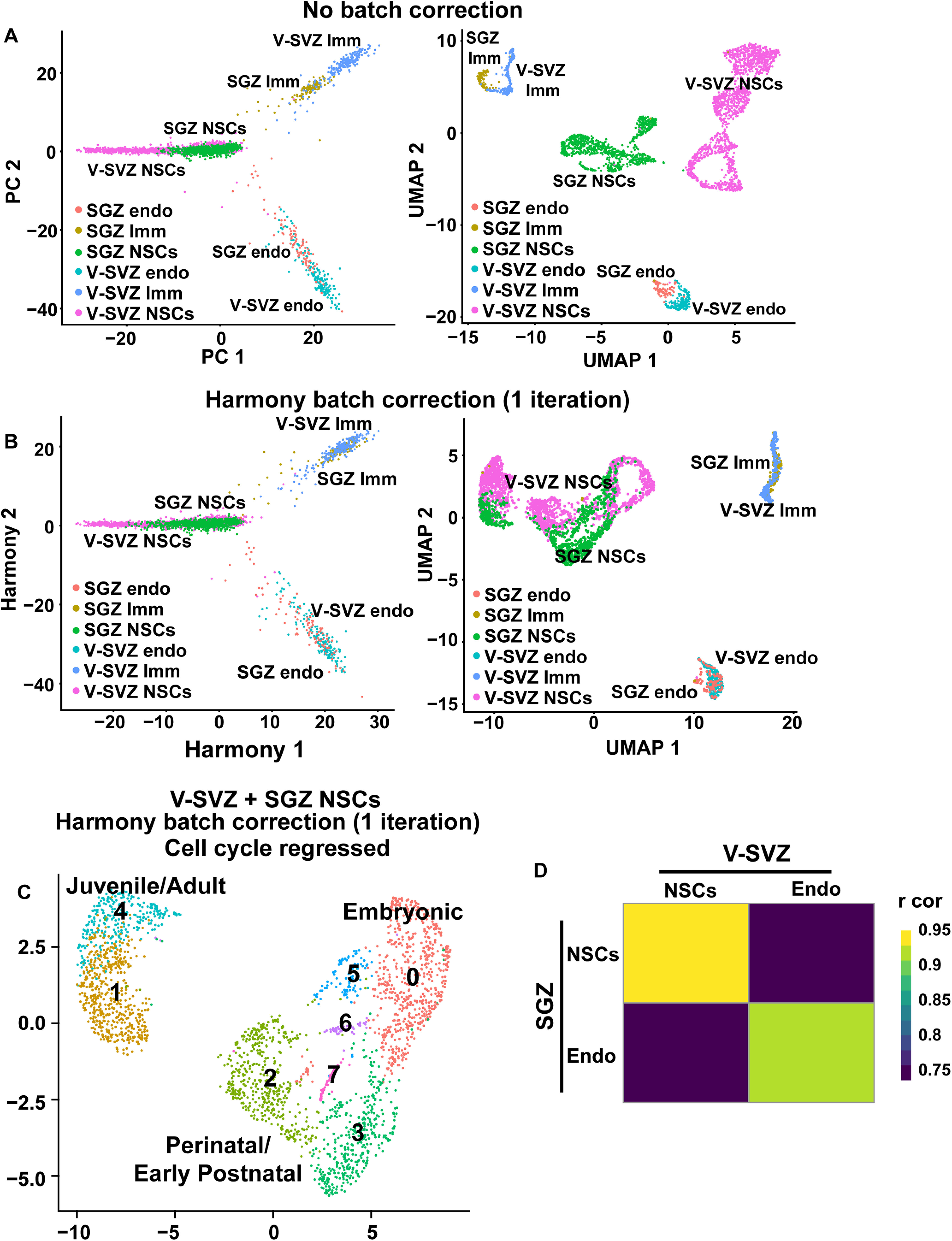
Batch correction and cell cycle regression for the combined V-SVZ and SGZ transcriptome analyses. ***A***, As a control to optimize the batch correction between V-SVZ and SGZ NSCs, raw transcriptomes from the dataset shown in Figure 3*B* were merged with endothelial cells from the P19 dentate gyrus (SGZ endo), endothelial cells from the P20 V-SVZ (V-SVZ endo), microglia from the P23 dentate gyrus (SGZ Imm), and microglia from the P20 V-SVZ (V-SVZ Imm). PCA visualization (left) and UMAP visualization (right) of the total dataset without batch correction showed that endothelial and immune cells from the two regions did not co-cluster well. Cells are colored based on cell type and region of origin. ***B***, The same dataset shown in ***A*** was batch corrected with one iteration of Harmony. The harmonized PCA visualization (left) and UMAP visualization (right) of the merged cells show that endothelial cells and immune cells were now well-clustered. Cells are colored based on cell type and region of origin. ***C***, Batch-corrected UMAP visualization of the merged V-SVZ NSC and SGZ NSC dataset shown in Figure 3*B*, where the cell cycle genes were regressed out as previously described (see Materials and Methods). Cells were grouped into color coded and numbered clusters based on gene expression profiles, and the NSCs of different ages are shown for direct comparison to Figure 3*B*. ***D***, Correlation heatmap showing Pearson correlation coefficients between averaged expression profiles of total NSCs (including all ages) and P19/20 V-SVZ and SGZ endothelial cells. Correlation coefficients are color coded as per the adjacent color key. Gene expression values were not batch-corrected.

One potential caveat of combining the two datasets is that the SGZ and V-SVZ cells were prepared and sequenced in two different laboratories using different protocols, and thus apparent differences might derive from batch effects as opposed to biological heterogeneity. To correct for this possibility, we also included endothelial cells (P19 SGZ and P20 V-SVZ) and microglial cells (P23 SGZ and P20 V-SVZ) from both datasets with the assumption that V-SVZ endothelial cells and microglia should be similar enough to co-cluster with the same cell types from the SGZ. However, when the combined dataset was visualized on a two-dimensional UMAP plot, the endothelial cells and microglia from the two different regions/datasets were partially segregated from each other (Fig. 4*A*), indicating variability because of batch effects. We therefore corrected for these batch effects using Harmony, a computational method for data integration that iteratively removes batch-mediated technical variation within principal component space of high dimensional data (Korsunsky et al., 2019; Tran et al., 2020). With the lowest level of Harmony correction, one iteration, there was complete integration of V-SVZ and SGZ endothelial and immune cells (Fig. 4*A*,*B*; see Materials and Methods).

Having established this protocol, we removed the endothelial and immune cells and analyzed only the RP/NSC transcriptomes, using one iteration of Harmony batch correction. UMAP visualization of these data (Fig. 3*B*) defined three groups of clusters, one including the juvenile and adult V-SVZ and SGZ NSCs, a second including the perinatal and postnatal NSCs of both origins and a third including the embryonic hippocampal, cortical and GE RPs. At any given developmental stage (adult, postnatal, or embryonic) there was some segregation between SGZ and V-SVZ NSCs suggesting that these two NSC populations were very similar but not identical (Fig. 3*B–D*).

One explanation for the differential clustering of developing and adult NSCs is that cell cycle genes associated with proliferation are partially responsible for driving this segregation. To test this idea, we removed 678 cell cycle-related genes (see Materials and Methods) and redid the analysis. UMAP visualization (Fig. 4*C*) showed that results were similar with and without removal of these cell cycle genes. There were three groups of clusters containing embryonic, perinatal/postnatal, or juvenile/adult NSCs, and there was some segregation of V-SVZ and SGZ NSCs of the same age within these clusters. Thus, cell cycle genes are not major drivers of the differential clustering seen for NSCs of different ages.

The strong age-dependent segregation of NSCs in the cluster plot (Fig. 3*B*) suggests that there may be greater transcriptional differences between NSCs at different developmental stages than there are between V-SVZ and SGZ precursors at the same time point. This conclusion was confirmed by performing two types of correlation analysis that do not involve any batch correction. The first was Pearson correlation analysis of average gene expression for V-SVZ and SGZ precursors at different timepoints (Fig. 3*E*). This analysis showed that at many timepoints, V-SVZ and SGZ precursors were more similar to each other than they were to any of the other precursor groups at different ages. For example, E14 V-SVZ and E16.5 SGZ RPs were correlated with a high value of *r* = 0.94, while E14 V-SVZ RPs and P20 V-SVZ NSCs were only correlated with *r* = 0.78. As predicted, all NSC populations were more similar to each other than they were to endothelial cells (Fig. 4*D*).

As a second approach, we performed a correlation analysis that compares single-cell transcriptomes rather than averaged gene expression (see Materials and Methods). To perform this single-cell correlation analysis, we first defined gene expression profiles for comparison to each individual cell transcriptome. As a first comparator, we determined average gene expression for E14 V-SVZ RPs versus juvenile/adult V-SVZ NSCs (P20/34/61; Fig. 3*F*, *x*-axis) and as a second comparator we determined average gene expression for E16.5 nonproliferative SGZ RPs versus juvenile/adult SGZ NSCs (P18–P132; Fig. 3*F*, *y*-axis). We then correlated all V-SVZ and SGZ NSC single-cell transcriptomes from all timepoints with these averaged datasets and used these correlations to assign a two-dimensional coordinate for each cell. This analysis, which uses gene expression values that are not batch corrected, showed that during embryogenesis and the first postnatal week, the V-SVZ and SGZ precursors were very similar, with the E16.5–P5 SGZ precursors closely mingled with the E17–P6/7 V-SVZ precursors of the same approximate age (Fig. 3*F*). By contrast, the juvenile/adult V-SVZ and SGZ NSCs were more similar to each other than they were to the developing precursors of the same origin (Fig. 3*F*). Thus, SGZ and V-SVZ precursors follow similar transcriptional trajectories from active embryonic RPs to dormant adult NSCs.

### Embryonic dentate gyrus and cortex RPs but not GE RPs express genes associated with excitatory neurogenesis and a common pallial origin

**Figure 5.**
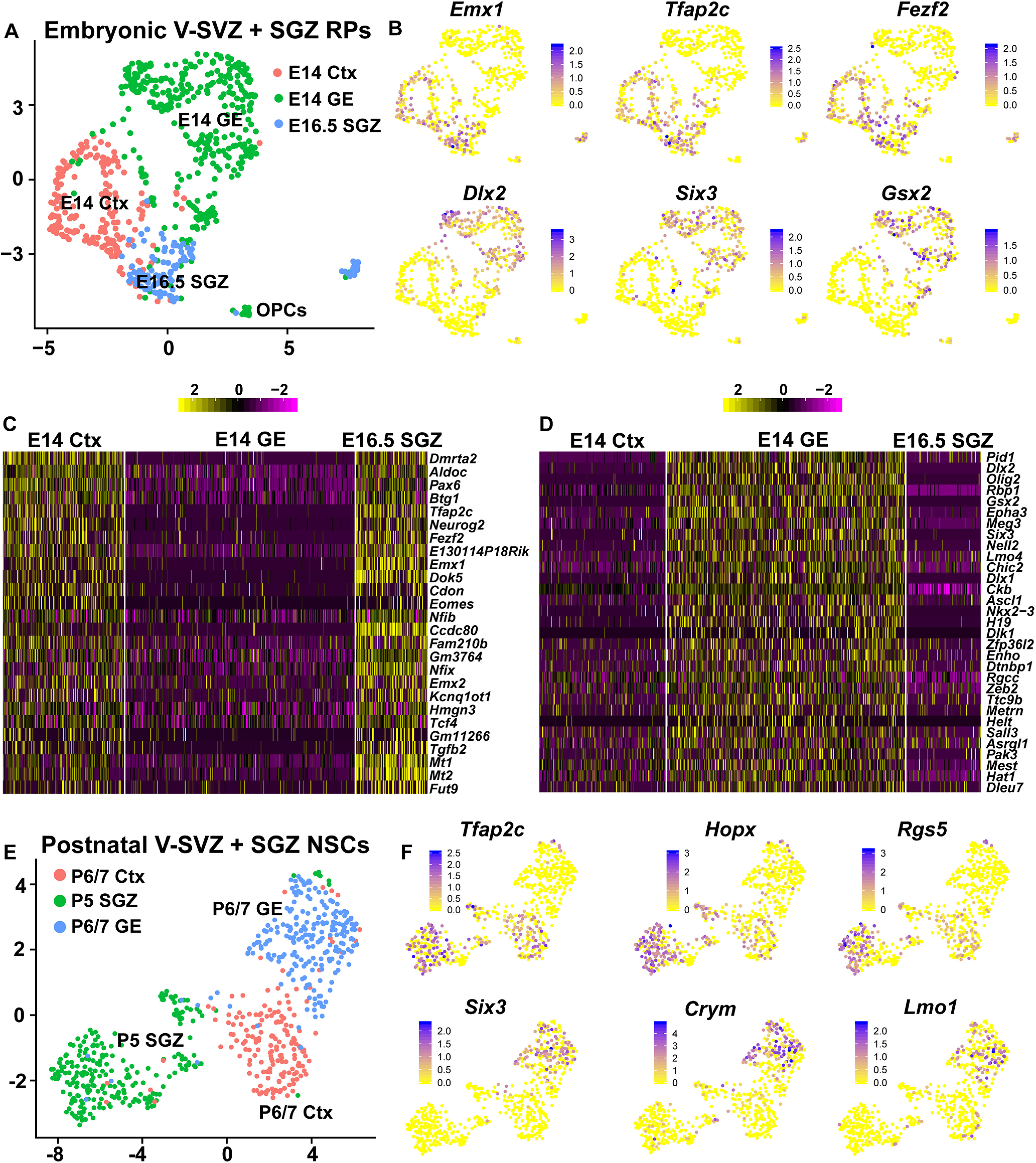
Embryonic dentate gyrus and cortex RPs express genes associated with excitatory neurogenesis and a common pallial origin. ***A***, Batch-corrected UMAP visualization of the transcriptomes of E16.5 dentate neuroepithelium RPs, E14 cortical RPs and E14 GE RPs, where transcriptomes are colored to indicate cell type. ***B***, UMAP marker gene expression overlays of the dataset in ***A***. Cells are color-coded for levels of gene expression as per the adjacent color keys. ***C***, ***D***, Heatmap illustrating common genes upregulated (***C***) or downregulated (***D***) in E14 cortical RPs and E16.5 SGZ RPs relative to E14 GE RPs. Genes are color-coded for levels of expression as per the adjacent color keys. Gene expression values are not batch-corrected. ***E***, Batch-corrected UMAP visualization of transcriptomes of P5 SGZ NSCs, P6/7 cortically derived V-SVZ NSCs and P6/7 GE-derived V-SVZ NSCs, colored to indicate cell type. ***F***, UMAP visualizations as in ***E***, overlaid for expression of genes defined in Borrett et al. (2020) as being enriched in cortical versus GE-derived V-SVZ NSCs. Cells are color-coded for levels of expression as per the adjacent color keys.

One explanation for the high similarity between SGZ and V-SVZ precursors is that they derive from RPs in adjacent lateral ventricle neuroepithelial regions during embryogenesis; dentate gyrus and cortical RPs are beside each other in the pallial region while GE RPs are immediately adjacent to cortical RPs in the subpallial region. We therefore directly compared E16.5 dentate gyrus RPs, E14 cortex RPs and E14 GE RPs, taking advantage of the fact that the V-SVZ cells were lineage traced so that cortex and GE-derived cells could be distinguished (see Borrett et al., 2020). We combined these different transcriptomes, put them through the pipeline together and used one round of Harmony batch correction. UMAP visualization of this combined dataset showed that the cortex, GE and dentate gyrus RP transcriptomes were largely but not completely segregated from each other (Fig. 5*A*), in good correspondence with the correlation analyses showing that these RPs were very similar to each other but not identical.

To more specifically identify differences between these RP populations, we focused on 117 genes that were previously-shown (Borrett et al., 2020) to be differentially expressed between cortical and GE RPs (average expression difference of ≥0.5; adj. *p* value < 0.05; FWER). Fifty-four of these genes were expressed at higher levels in cortical than GE RPs, and of these about half (26) were also significantly enriched in dentate gyrus versus GE RPs (Table 3), as shown by UMAP gene expression overlays (Fig. 5*B*) and by a heatmap indicating mRNA expression levels in single cells (Fig. 5*C*). These included genes like *Emx1*, *Tfap2c*, *Pax6*, *Fezf2*, *Neurog2*, and *Eomes*. Notably, some of these shared enriched genes are associated with glutamatergic neurogenesis (*Fezf2*, *Neurog2*, and *Eomes*), while others are associated with a pallial origin (*Emx1*, *Pax6*, and *Tfap2c*). We also asked about the other 63 genes, which were expressed at higher levels in GE versus cortical RPs (average expression difference of ≥0.5; adj. *p* value < 0.05; FWER). Of these, 49% were also higher in GE versus dentate gyrus RPs, as exemplified by *Dlx2*, *Six3*, and *Gsx2*, genes that are associated with GABAergic neurogenesis or GE identity (Fig. 5*B*,*D*; Table 3). Thus, the embryonic RP parents of V-SVZ and SGZ NSCs are all very similar to each, but are distinguished by expression of small cohorts of genes that are known to play important roles in determining regional identity and/or glutamatergic versus GABAergic neurogenesis.

**Table 3:** Differential gene expression analysis between E16.5 DG/cortex RPs and E14 GE RPs (related to Fig. 5)

DE gene	Average logFC	Adjusted *p* value
Dmrta2	0.63	6.00E-19
Aldoc	0.98	1.07E-15
Pax6	0.68	5.02E-13
Btg1	0.76	2.65E-18
Tfap2c	0.73	1.45E-27
Neurog2	1.08	6.34E-18
Fezf2	0.65	4.26E-18
E130114P18Rik	0.87	3.97E-22
Emx1	0.76	1.16E-29
Dok5	0.92	2.08E-37
Cdon	0.43	8.34E-04
Eomes	0.40	3.94E-02
Nfib	0.66	1.24E-14
Ccdc80	1.22	9.16E-45
Fam210b	0.64	7.33E-07
Gm3764	0.68	1.43E-14
Nfix	1.05	3.33E-23
Emx2	0.55	2.61E-04
Kcnq1ot1	0.55	1.90E-06
Hmgn3	0.65	2.46E-18
Tcf.4	0.56	3.15E-12
Gm11266	0.41	1.15E-06
Tgfb2	0.64	4.88E-19
Mt1	1.64	8.65E-38
Mt2	1.75	8.69E-29
Fut9	0.54	8.48E-10
Pid1	−0.56	1.30E-12
Dlx2	−1.20	4.90E-25
Olig2	−0.82	2.76E-15
Rbp1	−1.92	1.43E-38
Gsx2	−0.63	5.28E-14
Epha3	−0.77	2.77E-21
Meg3	−1.48	3.26E-26
Six3	−0.55	2.87E-13
Nell2	−0.67	5.69E-15
Lmo4	−0.43	6.56E-06
Chic2	−0.45	2.30E-07
Dlx1	−0.87	6.63E-16
Ckb	−1.11	4.64E-44
Ascl1	−0.73	9.94E-06
Nkx2-3	−0.50	2.08E-11
H19	−0.58	2.12E-11
Dlk1	−0.35	2.93E-04
Zfp36l2	−0.33	5.87E-05
Enho	−0.33	2.88E-04
Dtnbp1	−0.38	1.02E-05
Rgcc	−0.88	1.15E-17
Zeb2	−0.53	4.91E-10
Ttc9b	−0.29	2.04E-05
Metrn	−0.37	3.42E-05
Helt	−0.40	8.66E-03
Sall3	−0.36	2.27E-05
Asrgl1	−0.30	8.89E-03
Pak3	−0.51	1.74E-08
Mest	−0.95	5.72E-09
Hat1	−0.70	9.08E-15
Dleu7	−0.49	1.59E-05

A total of 117 genes was previously shown to be differentially expressed between E14 cortical RPs and E14 GE RPs (average difference > 0.5, FWER < 0.05) in Borrett et al. (2020). Of these, 54 were enriched in cortical RPs and 63 were enriched in GE RPs. These 117 genes were interrogated for their relative levels of expression in E16.5 DG RPs and E14 GE RPs (from the dataset shown in Fig. 5*A*). This analysis identified 26 (of 54) cortically enriched genes that were also significantly enriched in E16.5 SGZ RPs relative to E14 GE RPs, and 31 (of 63) GE-enriched genes that were also significantly enriched in E14 GE RPs relative to E16.5 SGZ RPs. These 57 genes are shown, as are the log fold changes in expression and adjusted *p* values (FWER < 0.05). Positive fold change values represent enriched expression in E16.5 SGZ RPs relative to E14 GE RPs and negative fold change values indicate enriched expression in E14 GE RPs relative to E16.5 SGZ RPs. These same 57 genes are depicted in the heatmaps in Figure 5*C,D*.

### Postnatal SGZ NSCs also express genes that may be associated with a pallial origin

We asked whether postnatal SGZ NSCs might continue to express genes reflective of their embryonic origin, as was previously seen for postnatal V-SVZ NSCs (Borrett et al., 2020). To ask this, we compared P5 SGZ NSCs to lineage-traced P6/7 V-SVZ NSCs deriving from the cortex and GE. We put all the transcriptomes through the batch-corrected pipeline together and visualized clustering on a UMAP (Fig. 5*E*). This analysis showed that as seen for the embryonic cells, NSCs from the cortex, GE, and dentate gyrus were largely segregated from one another. Together with the Pearson correlation analysis (Fig. 3*E*), these results indicate that these different postnatal NSC populations are very similar but not identical. We then asked about genes previously-defined as differentially expressed in cortically-derived versus GE-derived postnatal V-SVZ NSCs (Borrett et al., 2020). UMAP gene expression overlays showed that genes that were enriched in cortical NSCs such as *Hopx*, *Tfap2c*, and *Rgs5* were also enriched in dentate gyrus NSCs (Fig. 5*F*) and thus represented potential markers of their shared pallial origin. By contrast, genes that were enriched in GE NSCs and might be indicative of a subpallial origin, were largely not detectable in the SGZ NSCs, as exemplified by *Lmo1*, *Six3*, and *Crym* mRNAs (Fig. 5*F*). We validated one of the potential pallial NSC marker genes, *Rgs5*, by performing FISH on the P5 dentate gyrus. *Rgs5* mRNA was expressed in Sox2-positive SGZ cells that also expressed the precursor gene *Aldoc* (Fig. 1*E*), likely NSCs. Thus, as seen during embryogenesis, cortically-derived and dentate neuroepithelium-derived NSCs, but not GE-derived NSCs, express potential marker genes for a pallial origin.

### The developmental transition to a dormant adult NSC occurs over a prolonged postnatal period in the SGZ as it does in the V-SVZ

**Figure 6.**
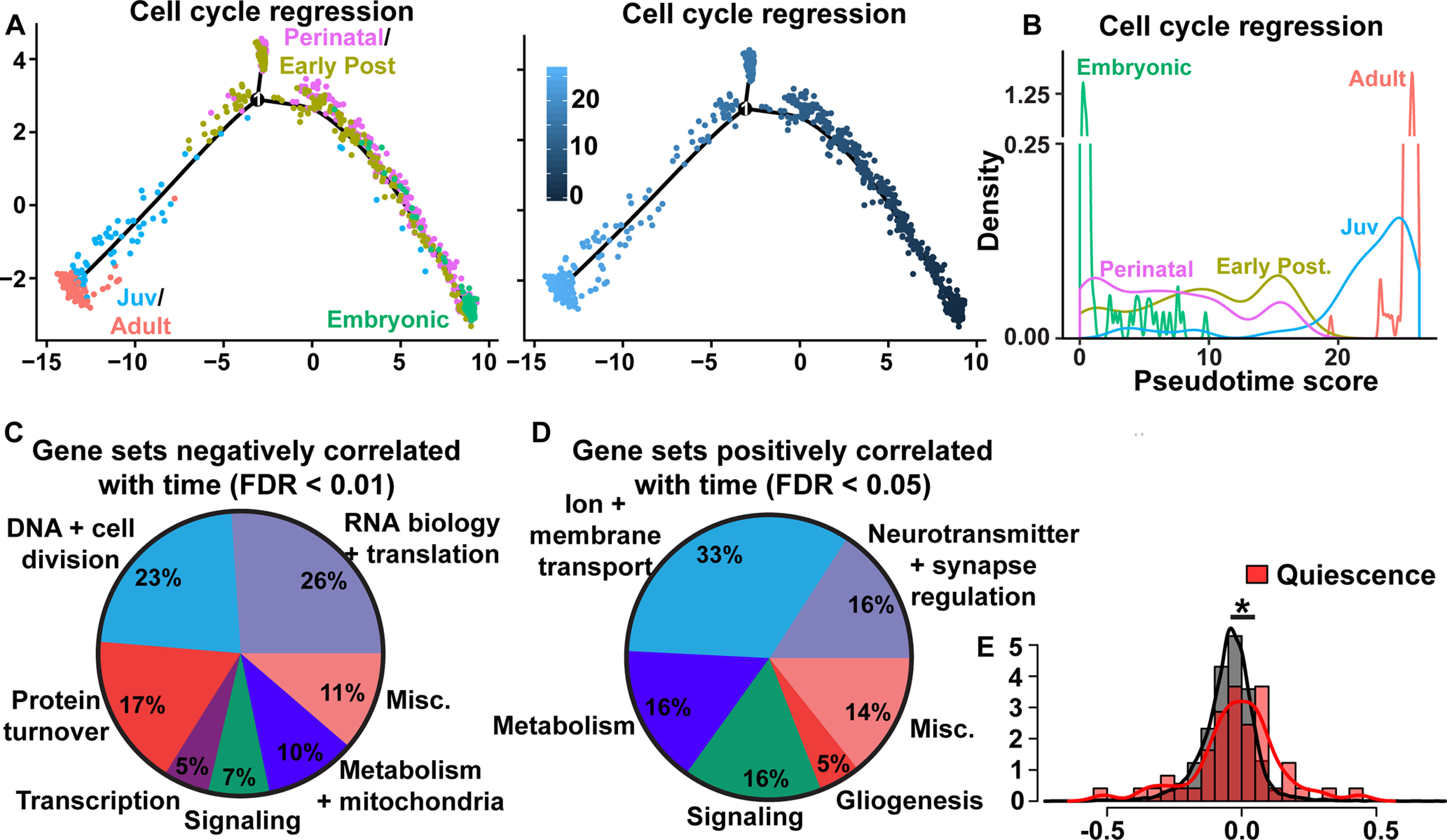
Mapping the trajectory from embryonic to adult SGZ NSCs with trajectory and GSEA analyses. ***A***, To understand the progression of SGZ NSCs from E16.5 to adulthood (P120/P132), SGZ NSCs at all ages (E16.5, P0, P5, P18, P19, P23, P120, P132) were ordered along a pseudotime trajectory using Monocle 2. To ensure cell cycle effects would not affect the ordering of the trajectory, we regressed out cell cycle genes (see Materials and Methods). SGZ NSCs along the trajectory are colored by age category (as defined in Fig. 3*A*, left) or by pseudotime ordering scores (right). These data are not batch corrected. ***B***, Density plot showing the relative distribution of pseudotime ordering scores of SGZ NSCs in the trajectory depicted in ***A*** from each age category. ***C***, ***D***, GSEA analysis of the combined SGZ NSC dataset from E16.5 to P132, performed without batch correction. Pie chart shows broad categories of genes sets negatively correlated (***C***) or positively correlated (***D***) with time that fell into a number of broad categories (FDR cutoffs are indicated). Categories negatively correlated with time in ***C*** include DNA replication, DNA repair, chromosome stability and segregation and the cell cycle (DNA + cell division), transcription, epigenetics, and chromatin regulation (Transcription), RNA homeostasis, translation and tRNA and ribosome biogenesis (RNA biology + translation), general protein processing and trafficking including ubiquitination and sumoylation (Protein turnover), signaling pathways (Signaling), and metabolism, oxidative phosphorylation and mitochondrial activity (Metabolism + mitochondria). Other categories are termed as miscellaneous (Misc.). The detailed categorization is shown in Table 4. Categories positively correlated with time (***D***) include neurotransmitter transport and synaptic regulation (Neurotransmitter + synapse regulation), Ion regulation and membrane transport (Ion + membrane transport), signaling pathways (Signaling), gliogenesis and metabolism and lipid oxidation (Metabolism). Other categories are termed as miscellaneous (Misc.). The detailed categorization is shown in Table 5. ***E***, Histogram of Spearman rank correlation coefficients of the combined SGZ NSC dataset for a signature of 49 quiescence genes described in Cheung and Rando (2013; red) versus all genes (gray). Correlations of >0 or <0 indicate expression increases or decreases over time; *p = 0.024, Wilcoxon rank-sum test.

In the V-SVZ, the transition from an active embryonic RP to a dormant postnatal NSC occurs over a prolonged, largely postnatal timeframe (Borrett et al., 2020). We asked whether this was also true for the SGZ using trajectory analysis, an approach that orders cells based on changes in their transcriptomes over pseudo-time. To do this, we combined transcriptomes of all dentate gyrus nonproliferative RP/NSCs from E16.5 to adulthood and performed a trajectory analysis using Monocle (Fig. 6*A*,*B*). We did not use batch correction for this analysis and, to ensure that the trajectory was not driven by precursor proliferative status, we removed the aforementioned 678 cell cycle-related genes. We also excluded a small number of cells (31 of 885 total) that expressed genes consistent with activated NSCs. This analysis resulted in a trajectory that correctly reflected the developmental progression. The E16.5 RPs were ordered at one end, and the adult dormant NSCs were at the other end. Some of the P0 and P5 NSCs were mingled with the E16.5 RPs, but most perinatal cells extended to eventually meet the juvenile NSCs, which then extended further along the trajectory to meet and mingle with the adult dormant NSCs at the other end. This trajectory was very similar to an analogous Monocle trajectory analysis of the V-SVZ RP/NSCs (Borrett et al., 2020), with the transition to an adult NSC state occurring gradually from birth until the third postnatal week.

These findings suggest that the transition from an active embryonic RP to a dormant adult NSC might be similar for the two major forebrain NSC populations. To further examine this idea and to determine what types of genes and/or cellular pathways are changed in SGZ NSCs at different ages, we performed a GSEA over SGZ developmental time from E16.5 to P132. We compared this GSEA to a previously-published (Borrett et al., 2020) analogous GSEA analysis for V-SVZ NSCs from E14 to P61. Notably, the SGZ analysis (Fig. 6*C*,*D*; Table 4) showed that 115 gene sets decreased significantly (adj. *p* value < 0.01; FDR) as the E16.5 SGZ RPs transitioned to dormant adult SGZ NSCs. Most of these gene sets involved basic cellular processes required to maintain an active, proliferative stem cell, including transcriptional programs required for cell division, DNA and chromosome replication, RNA biology, transcription, and protein synthesis and turnover, indicating that the predominant change that occurs over this timeframe is a transition to cellular dormancy. The developing NSCs were also enriched for gene sets involved with oxidative phosphorylation. Conversely, 63 gene sets were significantly enriched in dormant adult NSCs relative to their developing NSC counterparts (adj. *p* value < 0.05; FDR; Fig. 6*D*; Table 5). Notably, 65% of these were involved in regulating and/or sensing the niche environment, with a particular enrichment for sensing/handling neurotransmitters and ions like sodium and potassium. They also included gene sets involved in lipid metabolism and, of particular note, a quiescence gene set (Fig. 6*E*) that was shown to be significantly enriched as V-SVZ NSCs transitioned to dormancy (Borrett et al., 2020). Thus, as previously shown for adult V-SVZ NSCs, adult SGZ NSCs are transcriptionally quiet with regard to genes involved in maintaining an active state and instead selectively express gene sets that allow them to sense and maintain themselves in a dynamic neuronal environment and to perform lipid metabolism.

**Table 4:** Gene sets negatively correlated with time, as analyzed by GSEA for total SGZ NSCs from E16.5 to P132 (related to Fig. 6)

Pathway names	Adj. *p* value (FDR)	Norm. Enr. Score	nMore- Extreme	Size	Category
CYTOPLASMIC RIBOSOMAL PROTEINS%WIKIPATHWAYS_20191210%WP163%MUS MUSCULUS	2.29E-03	−2.73	0	80	RNA translation + ribosomes
FORMATION OF A POOL OF FREE 40S SUBUNITS%REACTOME DATABASE ID RELEASE 71%72689	2.29E-03	−2.59	0	51	RNA translation + ribosomes
VIRAL MRNA TRANSLATION%REACTOME DATABASE ID RELEASE 71%192823	2.29E-03	−2.57	0	39	IR
L13A-MEDIATED TRANSLATIONAL SILENCING OF CERULOPLASMIN EXPRESSION%REACTOME DATABASE ID RELEASE 71%156827	2.29E-03	−2.57	0	61	Miscellaneous
EUKARYOTIC TRANSLATION ELONGATION%REACTOME%R-HSA-156842.2	2.29E-03	−2.57	0	42	RNA translation + ribosomes
NONSENSE MEDIATED DECAY (NMD) INDEPENDENT OF THE EXON JUNCTION COMPLEX (EJC)%REACTOME%R-HSA-975956.1	2.29E-03	−2.56	0	45	DNA + cell cycle
SELENOCYSTEINE SYNTHESIS%REACTOME DATABASE ID RELEASE 71%2408557	2.29E-03	−2.55	0	43	Miscellaneous
REGULATION OF EXPRESSION OF SLITS AND ROBOS%REACTOME DATABASE ID RELEASE 71%9010553	2.29E-03	−2.54	0	111	Signaling
EUKARYOTIC TRANSLATION TERMINATION%REACTOME%R-HSA-72764.4	2.29E-03	−2.53	0	43	RNA translation + ribosomes
RESPONSE OF EIF2AK4 (GCN2) TO AMINO ACID DEFICIENCY%REACTOME DATABASE ID RELEASE 71%9633012	2.29E-03	−2.51	0	50	RNA translation + ribosomes
SRP-DEPENDENT COTRANSLATIONAL PROTEIN TARGETING TO MEMBRANE%REACTOME%R-HSA-1799339.2	2.29E-03	−2.48	0	61	Protein processing
ACTIVATION OF THE MRNA UPON BINDING OF THE CAP-BINDING COMPLEX AND EIFS, AND SUBSEQUENT BINDING TO 43S%REACTOME%R-HSA-72662.3	2.29E-03	−2.42	0	43	RNA translation + ribosomes
HALLMARK_MYC_TARGETS_V1%MSIGDB_C2%HALLMARK_MYC_TARGETS_V1	2.29E-03	−2.40	0	184	DNA + cell cycle
SELENOAMINO ACID METABOLISM%REACTOME DATABASE ID RELEASE 71%2408522	2.29E-03	−2.39	0	62	Metabolism
AUF1 (HNRNP D0) BINDS AND DESTABILIZES MRNA%REACTOME DATABASE ID RELEASE 71%450408	2.29E-03	−2.39	0	49	RNA translation + ribosomes
REGULATION OF ORNITHINE DECARBOXYLASE (ODC)%REACTOME%R-HSA-350562.2	2.29E-03	−2.37	0	48	Miscellaneous
PROTEASOME DEGRADATION%WIKIPATHWAYS_20191210%WP519%MUS MUSCULUS	2.29E-03	−2.35	0	50	Protein processing
THE ROLE OF GTSE1 IN G2 M PROGRESSION AFTER G2 CHECKPOINT%REACTOME DATABASE ID RELEASE 71%8852276	2.29E-03	−2.35	0	53	DNA + cell cycle
APC C:CDC20 MEDIATED DEGRADATION OF SECURIN%REACTOME%R-HSA-174154.2	2.29E-03	−2.34	0	61	IR
CYTOPLASMIC TRANSLATION%GOBP%GO:0002181	2.29E-03	−2.33	0	61	RNA translation + ribosomes
ER-PHAGOSOME PATHWAY%REACTOME DATABASE ID RELEASE 71%1236974	2.29E-03	−2.31	0	69	Protein processing
SCF(SKP2)-MEDIATED DEGRADATION OF P27 P21%REACTOME%R-HSA-187577.3	2.29E-03	−2.29	0	56	DNA + cell cycle
INFLUENZA VIRAL RNA TRANSCRIPTION AND REPLICATION%REACTOME DATABASE ID RELEASE 71%168273	2.29E-03	−2.27	0	80	IR
INFLUENZA INFECTION%REACTOME%R-HSA-168254.2	2.29E-03	−2.22	0	96	IR
ASSEMBLY OF THE PRE-REPLICATIVE COMPLEX%REACTOME%R-HSA-68867.4	2.29E-03	−2.20	0	63	DNA + cell cycle
REGULATION OF MITOTIC CELL CYCLE%REACTOME%R-HSA-453276.2	2.29E-03	−2.20	0	78	DNA + cell cycle
REGULATION OF MRNA STABILITY BY PROTEINS THAT BIND AU-RICH ELEMENTS%REACTOME%R-HSA-450531.4	2.29E-03	−2.19	0	77	RNA translation + ribosomes
MAJOR PATHWAY OF RRNA PROCESSING IN THE NUCLEOLUS AND CYTOSOL%REACTOME%R-HSA-6791226.3	2.29E-03	−2.19	0	124	RNA translation + ribosomes
SWITCHING OF ORIGINS TO A POST-REPLICATIVE STATE%REACTOME DATABASE ID RELEASE 71%69052	2.29E-03	−2.15	0	84	DNA + cell cycle
RUNX1 REGULATES TRANSCRIPTION OF GENES INVOLVED IN DIFFERENTIATION OF HSCS%REACTOME%R-HSA-8939236.1	2.29E-03	−2.14	0	72	IR
MITOCHONDRIAL TRANSLATION ELONGATION%REACTOME%R-HSA-5389840.1	2.29E-03	−2.13	0	83	RNA translation + ribosomes
HOST INTERACTIONS OF HIV FACTORS%REACTOME%R-HSA-162909.1	2.29E-03	−2.13	0	110	IR
RIBOSOMAL LARGE SUBUNIT BIOGENESIS%GOBP%GO:0042273	2.29E-03	−2.12	0	66	RNA translation + ribosomes
NEGATIVE REGULATION OF RNA SPLICING%GOBP%GO:0033119	2.29E-03	−2.10	0	28	RNA translation + ribosomes
MRNA SPLICING%REACTOME%R-HSA-72172.3	2.29E-03	−2.09	0	176	RNA translation + ribosomes
RIBOSOME ASSEMBLY%GOBP%GO:0042255	2.29E-03	−2.09	0	57	RNA translation + ribosomes
RRNA PROCESSING%GOBP%GO:0006364	2.29E-03	−2.09	0	148	RNA translation + ribosomes
SYNTHESIS OF DNA%REACTOME DATABASE ID RELEASE 71%69239	2.29E-03	−2.07	0	113	DNA + cell cycle
COOPERATION OF PREFOLDIN AND TRIC CCT IN ACTIN AND TUBULIN FOLDING%REACTOME%R-HSA-389958.2	2.29E-03	−2.05	0	24	Miscellaneous
UBIQUITIN PROTEASOME PATHWAY%PANTHER PATHWAY%P00060	2.29E-03	−2.05	0	39	Protein processing
TRANSLATION FACTORS%WIKIPATHWAYS_20191210%WP307%MUS MUSCULUS	2.29E-03	−2.00	0	47	RNA translation + ribosomes
RIBOSOMAL SMALL SUBUNIT BIOGENESIS%GOBP%GO:0042274	2.29E-03	−2.00	0	63	RNA translation + ribosomes
RNA SPLICING%GOBP%GO:0008380	2.29E-03	−1.99	0	198	RNA processing + translation
HALLMARK_OXIDATIVE_PHOSPHORYLATION%MSIGDB_C2%HALLMARK_OXIDATIVE_PHOSPHORYLATION	2.29E-03	−1.97	0	178	Metabolism: Ox-Phos
RIBONUCLEOPROTEIN COMPLEX ASSEMBLY%GOBP%GO:0022618	2.29E-03	−1.94	0	187	RNA processing + translation
PID_MYC_ACTIV_PATHWAY%MSIGDB_C2%PID_MYC_ACTIV_PATHWAY	2.29E-03	−1.93	0	66	DNA + cell cycle
PROGRAMMED CELL DEATH%REACTOME%R-HSA-5357801.2	2.29E-03	−1.93	0	139	Miscellaneous
PROTEIN REFOLDING%GOBP%GO:0042026	2.29E-03	−1.92	0	23	Protein processing
G2 M CHECKPOINTS%REACTOME%R-HSA-69481.3	2.29E-03	−1.92	0	122	DNA + cell cycle
TOXIN TRANSPORT%GOBP%GO:1901998	2.29E-03	−1.92	0	34	Miscellaneous
PROTEIN FOLDING%GOBP%GO:0006457	2.29E-03	−1.91	0	101	Protein processing
HALLMARK_E2F_TARGETS%MSIGDB_C2%HALLMARK_E2F_TARGETS	2.29E-03	−1.91	0	193	DNA + cell cycle
MITOTIC ANAPHASE%REACTOME DATABASE ID RELEASE 71%68882	2.29E-03	−1.90	0	161	DNA + cell cycle
PROTEIN LOCALIZATION TO MITOCHONDRION%GOBP%GO:0070585	2.29E-03	−1.90	0	67	Protein processing
G1 S TRANSITION%REACTOME DATABASE ID RELEASE 71%69206	2.29E-03	−1.89	0	123	DNA + cell cycle
TRANSCRIPTIONAL REGULATION BY RUNX1%REACTOME%R-HSA-8878171.3	2.29E-03	−1.88	0	156	Transcriptional regulation
NEGATIVE REGULATION OF MRNA METABOLIC PROCESS%GOBP%GO:1903312	2.29E-03	−1.88	0	72	RNA processing + translation
RNA POLYMERASE II TRANSCRIPTION TERMINATION%REACTOME%R-HSA-73856.4	2.29E-03	−1.87	0	55	Transcriptional regulation
G2 M TRANSITION%REACTOME%R-HSA-69275.5	2.29E-03	−1.86	0	164	DNA + cell cycle
NUCLEOSIDE TRIPHOSPHATE BIOSYNTHETIC PROCESS%GOBP%GO:0009142	2.29E-03	−1.86	0	62	DNA + cell cycle
GENE AND PROTEIN EXPRESSION BY JAK-STAT SIGNALING AFTER INTERLEUKIN-12 STIMULATION%REACTOME%R-HSA-8950505.3	2.29E-03	−1.84	0	27	Signaling
EUKARYOTIC TRANSCRIPTION INITIATION%WIKIPATHWAYS_20191210%WP567%MUS MUSCULUS	2.29E-03	−1.83	0	41	Transcriptional regulation
NADH DEHYDROGENASE COMPLEX ASSEMBLY%GOBP%GO:0010257	2.29E-03	−1.82	0	45	Metabolism: Ox-Phos
RIBONUCLEOPROTEIN COMPLEX LOCALIZATION%GOBP%GO:0071166	2.29E-03	−1.82	0	54	RNA processing + translation
RNA LOCALIZATION%GOBP%GO:0006403	2.29E-03	−1.81	0	104	RNA processing + translation
PROTEIN TRANSMEMBRANE TRANSPORT%GOBP%GO:0071806	2.29E-03	−1.81	0	51	Protein processing
TAT-MEDIATED ELONGATION OF THE HIV-1 TRANSCRIPT%REACTOME DATABASE ID RELEASE 71%167246	2.29E-03	−1.80	0	40	IR
HALLMARK_MTORC1_SIGNALING%MSIGDB_C2%HALLMARK_MTORC1_SIGNALING	2.29E-03	−1.80	0	188	Signaling
ELECTRON TRANSPORT CHAIN%WIKIPATHWAYS_20191210%WP295%MUS MUSCULUS	2.29E-03	−1.79	0	94	Metabolism: Ox-Phos
NUCLEOTIDE EXCISION REPAIR%REACTOME DATABASE ID RELEASE 71%5696398	2.29E-03	−1.78	0	101	DNA + cell cycle
MITOCHONDRIAL RESPIRATORY CHAIN COMPLEX ASSEMBLY%GOBP%GO:0033108	2.29E-03	−1.77	0	75	Metabolism: Ox-Phos
REGULATION OF MRNA SPLICING, VIA SPLICEOSOME%GOBP%GO:0048024	2.29E-03	−1.76	0	92	RNA processing + translation
POSITIVE REGULATION OF VIRAL GENOME REPLICATION%GOBP%GO:0045070	2.29E-03	−1.76	0	30	IR
TNF-ALPHA NF-KB SIGNALING PATHWAY%WIKIPATHWAYS_20191210%WP246%MUS MUSCULUS	2.29E-03	−1.75	0	165	Signaling
OXIDATIVE PHOSPHORYLATION%WIKIPATHWAYS_20191210%WP1248%MUS MUSCULUS	2.29E-03	−1.75	0	57	Metabolism: Ox-Phos
REGULATION OF UBIQUITIN-PROTEIN TRANSFERASE ACTIVITY%GOBP%GO:0051438	2.29E-03	−1.73	0	45	Protein processing
MITOCHONDRIAL GENE EXPRESSION%GOBP%GO:0140053	2.29E-03	−1.73	0	75	Transcriptional regulation
HALLMARK_DNA_REPAIR%MSIGDB_C2%HALLMARK_DNA_REPAIR	2.29E-03	−1.71	0	143	DNA + cell cycle
HALLMARK_UNFOLDED_PROTEIN_RESPONSE%MSIGDB_C2%HALLMARK_UNFOLDED_PROTEIN_RESPONSE	2.29E-03	−1.70	0	108	Protein processing
HIV LIFE CYCLE%REACTOME%R-HSA-162587.2	2.29E-03	−1.69	0	132	IR
CELLULAR RESPONSE TO HEAT STRESS%REACTOME%R-HSA-3371556.1	2.29E-03	−1.69	0	79	Miscellaneous
THE CITRIC ACID (TCA) CYCLE AND RESPIRATORY ELECTRON TRANSPORT%REACTOME%R-HSA-1428517.1	2.29E-03	−1.68	0	149	Metabolism: Ox-Phos
ATP METABOLIC PROCESS%GOBP%GO:0046034	2.29E-03	−1.67	0	121	Metabolism
NUCLEOSIDE TRIPHOSPHATE METABOLIC PROCESS%GOBP%GO:0009141	2.29E-03	−1.67	0	161	DNA + cell cycle
HALLMARK_G2M_CHECKPOINT%MSIGDB_C2%HALLMARK_G2M_CHECKPOINT	2.29E-03	−1.66	0	180	DNA + cell cycle
PROTEIN-DNA COMPLEX SUBUNIT ORGANIZATION%GOBP%GO:0071824	2.29E-03	−1.62	0	127	Transcriptional regulation
NUCLEOSIDE MONOPHOSPHATE METABOLIC PROCESS%GOBP%GO:0009123	2.29E-03	−1.62	0	150	DNA + cell cycle
PROTEIN STABILIZATION%GOBP%GO:0050821	2.29E-03	−1.59	0	156	Protein processing
CILIUM ASSEMBLY%REACTOME DATABASE ID RELEASE 71%5617833	2.29E-03	−1.58	0	169	Miscellaneous
PROTEIN TARGETING%GOBP%GO:0006605	2.29E-03	−1.58	0	183	Protein processing
METHYLATION%GOBP%GO:0032259	2.29E-03	−1.51	0	191	Miscellaneous
HALLMARK_GLYCOLYSIS%MSIGDB_C2%HALLMARK_GLYCOLYSIS	2.29E-03	−1.49	0	172	Metabolism
PROTEASOMAL UBIQUITIN-INDEPENDENT PROTEIN CATABOLIC PROCESS%GOBP%GO:0010499	2.30E-03	−2.27	0	21	Protein processing
ATP SYNTHESIS COUPLED PROTON TRANSPORT%GOBP%GO:0015986	2.30E-03	−2.08	0	16	Metabolism: Ox-Phos
CELLULAR RESPONSE TO INTERLEUKIN-7%GOBP%GO:0098761	2.30E-03	−2.07	0	16	Signaling
FORMATION OF TUBULIN FOLDING INTERMEDIATES BY CCT TRIC%REACTOME%R-HSA-389960.2	2.30E-03	−2.03	0	17	Miscellaneous
CELLULAR RESPONSE TO INTERLEUKIN-4%GOBP%GO:0071353	2.30E-03	−1.94	0	18	Signaling
SPERM-EGG RECOGNITION%GOBP%GO:0035036	2.30E-03	−1.97	0	15	IR
ER TO GOLGI VESICLE-MEDIATED TRANSPORT%GOBP%GO:0006888	3.91E-03	−1.61	1	93	Protein processing
HALLMARK_MYC_TARGETS_V2%MSIGDB_C2%HALLMARK_MYC_TARGETS_V2	3.95E-03	−1.75	1	56	DNA + cell cycle
REGULATION OF SIGNAL TRANSDUCTION BY P53 CLASS MEDIATOR%GOBP%GO:1901796	3.95E-03	−1.72	1	58	Signaling
CELLULAR RESPONSE TO HEAT%GOBP%GO:0034605	3.98E-03	−1.69	1	46	Miscellaneous
CRISTAE FORMATION%REACTOME DATABASE ID RELEASE 71%8949613	4.17E-03	−1.83	1	24	Miscellaneous
RIBOSOMAL SMALL SUBUNIT ASSEMBLY%GOBP%GO:0000028	4.18E-03	−1.89	1	18	RNA processing + translation
PROTEIN PEPTIDYL-PROLYL ISOMERIZATION%GOBP%GO:0000413	4.18E-03	−1.83	1	18	Protein processing
POSITIVE REGULATION OF TRANSCRIPTION INITIATION FROM RNA POLYMERASE II PROMOTER%GOBP%GO:0060261	4.18E-03	−1.81	1	20	Transcriptional regulation
VIRAL GENE EXPRESSION%GOBP%GO:0019080	4.18E-03	−1.81	1	20	IR
BBSOME-MEDIATED CARGO-TARGETING TO CILIUM%REACTOME DATABASE ID RELEASE 71%5620922	4.18E-03	−1.79	1	21	Miscellaneous
MITOTIC PROMETAPHASE%REACTOME%R-HSA-68877.5	5.40E-03	−1.48	2	167	DNA + cell cycle
POSITIVE REGULATION OF TRANSLATION%GOBP%GO:0045727	5.40E-03	−1.62	2	104	RNA processing + translation
DNA REPLICATION%GOBP%GO:0006260	5.40E-03	−1.57	2	117	DNA + cell cycle
RNA CATABOLIC PROCESS%GOBP%GO:0006401	5.40E-03	−1.54	2	112	RNA processing + translation
MITOCHONDRIAL PROTEIN IMPORT%REACTOME DATABASE ID RELEASE 71%1268020	5.50E-03	−1.69	2	57	Miscellaneous
DEADENYLATION-DEPENDENT MRNA DECAY%REACTOME DATABASE ID RELEASE 71%429914	5.50E-03	−1.69	2	55	RNA processing + translation
NEGATIVE REGULATION OF PROTEIN POLYMERIZATION%GOBP%GO:0032272	5.52E-03	−1.67	2	52	Protein processing
RNA MODIFICATION%GOBP%GO:0009451	6.86E-03	−1.55	3	114	RNA processing + translation
DNA RECOMBINATION%GOBP%GO:0006310	6.86E-03	−1.53	3	142	DNA + cell cycle
REGULATION OF NUCLEAR DIVISION%GOBP%GO:0051783	6.86E-03	−1.53	3	144	DNA + cell cycle
INTERSPECIES INTERACTION BETWEEN ORGANISMS%GOBP%GO:0044419	6.86E-03	−1.46	3	173	IR
CHROMOSOME MAINTENANCE%REACTOME%R-HSA-73886.2	6.97E-03	−1.65	3	72	DNA + cell cycle
SIG_REGULATION_OF_THE_ACTIN_CYTOSKELETON_BY_RHO_GTPASES%MSIGDB_C2%SIG_REGULATION_OF_THE_ACTIN_CYTOSKELETON_BY_RHO_GTPASES	7.29E-03	−1.73	3	28	Signaling
NEGATIVE REGULATION OF UBIQUITIN-PROTEIN TRANSFERASE ACTIVITY%GOBP%GO:0051444	7.60E-03	−1.76	3	16	Protein processing
TRNA METABOLIC PROCESS%GOBP%GO:0006399	8.03E-03	−1.51	4	137	RNA processing + translation
REGULATION OF TP53 ACTIVITY%REACTOME%R-HSA-5633007.3	9.43E-03	−1.50	5	138	Miscellaneous
AUTOPHAGY%REACTOME%R-HSA-9612973.1	9.46E-03	−1.52	5	107	Miscellaneous
NEGATIVE REGULATION OF PROTEOLYSIS INVOLVED IN CELLULAR PROTEIN CATABOLIC PROCESS%GOBP%GO:1903051	9.65E-03	−1.62	5	67	Protein processing

Shown are gene sets that are negatively correlated with time (decreasing in the transition from embryonic RPs to adult NSCs) where FDR < 0.01, analyzed from the combined SGZ RP/NSC dataset (a total of 885 cells, highlighted in blue in Fig. 3*C*). Also shown are the adjusted *p* values (adj. *p* value; FDR), enrichment scores (Norm. Enr. score), the size of the gene set and the number of times a random gene set had a more extreme enrichment score than the gene set (nMoreExtreme). Gene sets are ordered from most to least significant from top to bottom. These gene sets were also categorized with regard to a number of broad categories, including DNA replication, DNA repair, chromosome stability and segregation and the cell cycle (DNA + cell cycle), transcription, epigenetics and chromatin regulation (transcriptional regulation), RNA homeostasis, translation and tRNA and ribosome biogenesis (RNA processing + translation), general protein processing and trafficking including ubiquitination and sumoylation (protein processing), signaling pathways (signaling), and metabolism, oxidative phosphorylation and mitochondrial activity (metabolism). Other categories are termed as miscellaneous (misc.) and irrelevant gene sets are termed as IR.

**Table 5:** Gene sets positively correlated with time, as analyzed by GSEA for total SGZ NSCs from E16.5 to P132 (related to Fig. 6)

Pathway	Adj. *p* value (FDR)	Norm. Enr. score	nMore- Extreme	Size	Category
IONOTROPIC GLUTAMATE RECEPTOR SIGNALING PATHWAY%GOBP%GO:0035235	9.84E-03	2.46	0	15	Neurotransmitter/synaptic regulation
CELLULAR RESPONSE TO STEROL%GOBP%GO:0036315	9.84E-03	2.46	0	15	Signaling
NEUROTRANSMITTER UPTAKE%GOBP%GO:0001504	1.01E-02	2.49	0	16	Neurotransmitter/synaptic regulation
EXPORT ACROSS PLASMA MEMBRANE%GOBP%GO:0140115	1.01E-02	2.23	0	16	Membrane transport + ion balance
REGULATION OF MEMBRANE PROTEIN ECTODOMAIN PROTEOLYSIS%GOBP%GO:0051043	1.03E-02	2.32	0	17	Miscellaneous
NEGATIVE REGULATION OF PEPTIDYL-THREONINE PHOSPHORYLATION%GOBP%GO:0010801	1.03E-02	2.21	0	17	Miscellaneous
ACIDIC AMINO ACID TRANSPORT%GOBP%GO:0015800	1.30E-02	2.75	0	27	Membrane transport + ion balance
REGULATION OF TRIGLYCERIDE METABOLIC PROCESS%GOBP%GO:0090207	1.37E-02	2.22	0	29	Metabolism (lipid)
SODIUM ION TRANSMEMBRANE TRANSPORT%GOBP%GO:0035725	1.41E-02	2.43	0	30	Membrane transport + ion balance
IONOTROPIC GLUTAMATE RECEPTOR PATHWAY%PANTHER PATHWAY%P00037	1.49E-02	2.43	0	32	Neurotransmitter/synaptic regulation
NEUROTRANSMITTER RELEASE CYCLE%REACTOME%R-HSA-112310.5	1.51E-02	2.46	0	34	Neurotransmitter/synaptic regulation
RESPONSE TO DIETARY EXCESS%GOBP%GO:0002021	1.68E-02	2.25	1	15	IR
POSITIVE REGULATION OF ANION TRANSPORT%GOBP%GO:1903793	1.76E-02	2.29	0	39	Membrane transport + ion balance
ZINC ION HOMEOSTASIS%GOBP%GO:0055069	1.78E-02	2.09	1	18	Membrane transport + ion balance
SYNAPTIC_VESICLE_TRAFFICKING%PANTHER PATHWAY%P05734	1.78E-02	2.09	1	18	Neurotransmitter/synaptic regulation
EXPLORATION BEHAVIOR%GOBP%GO:0035640	1.95E-02	2.01	1	23	IR
REGULATION OF BONE RESORPTION%GOBP%GO:0045124	1.95E-02	2.00	1	23	IR
MATING%GOBP%GO:0007618	1.95E-02	1.94	1	23	IR
CYTOKINE SECRETION%GOBP%GO:0050663	1.95E-02	1.92	1	23	Membrane transport + ion balance
REGULATION OF SYNAPTIC TRANSMISSION, GLUTAMATERGIC%GOBP%GO:0051966	2.05E-02	2.14	0	49	Neurotransmitter/synaptic regulation
IMPORT ACROSS PLASMA MEMBRANE%GOBP%GO:0098739	2.32E-02	2.35	0	53	Membrane transport + ion balance
NEUROMUSCULAR PROCESS CONTROLLING BALANCE%GOBP%GO:0050885	2.32E-02	1.92	0	53	IR
EFFECTS OF PIP2 HYDROLYSIS%REACTOME%R-HSA-114508.2	2.41E-02	1.94	2	19	Metabolism (lipid)
OLIGODENDROCYTE DIFFERENTIATION%GOBP%GO:0048709	2.42E-02	2.20	0	56	Gliogenesis
POSITIVE REGULATION OF AMINE TRANSPORT%GOBP%GO:0051954	2.48E-02	2.17	1	34	Membrane transport + ion balance
POSTSYNAPTIC MEMBRANE ORGANIZATION%GOBP%GO:0001941	2.57E-02	1.89	2	22	Neurotransmitter/synaptic regulation
TRANSPORT OF INORGANIC CATIONS ANIONS AND AMINO ACIDS OLIGOPEPTIDES%REACTOME%R-HSA-425393.2	2.64E-02	2.38	0	62	Membrane transport + ion balance
ION HOMEOSTASIS%REACTOME%R-HSA-5578775.1	2.64E-02	1.93	1	36	Membrane transport + ion balance
ION TRANSPORT BY P-TYPE ATPASES%REACTOME DATABASE ID RELEASE 71%936837	2.64E-02	1.89	1	36	Membrane transport + ion balance
POSITIVE REGULATION OF LIPID BIOSYNTHETIC PROCESS%GOBP%GO:0046889	2.67E-02	2.12	0	63	Metabolism (lipid)
REGULATION OF COMPLEMENT CASCADE%REACTOME DATABASE ID RELEASE 71%977606	2.70E-02	1.98	3	15	Signaling
REGULATION OF GLYCOPROTEIN METABOLIC PROCESS%GOBP%GO:1903018	2.71E-02	2.25	1	37	Metabolism
REPRODUCTIVE BEHAVIOR%GOBP%GO:0019098	2.74E-02	1.99	2	26	IR
INTERACTION BETWEEN L1 AND ANKYRINS%REACTOME%R-HSA-445095.1	2.82E-02	1.95	3	17	Miscellaneous
RESPONSE TO COPPER ION%GOBP%GO:0046688	2.82E-02	1.89	3	17	Membrane transport + ion balance
CELLULAR POTASSIUM ION TRANSPORT%GOBP%GO:0071804	2.83E-02	2.56	0	67	Membrane transport + ion balance
PROTEIN-PROTEIN INTERACTIONS AT SYNAPSES%REACTOME DATABASE ID RELEASE 71%6794362	2.83E-02	2.04	0	67	Neurotransmitter/synaptic regulation
LONG-CHAIN FATTY ACID METABOLIC PROCESS%GOBP%GO:0001676	2.88E-02	2.19	1	40	Metabolism (lipid)
ADENYLATE CYCLASE-INHIBITING G PROTEIN-COUPLED RECEPTOR SIGNALING PATHWAY%GOBP%GO:0007193	2.90E-02	1.95	2	29	Signaling
PID_UPA_UPAR_PATHWAY%MSIGDB_C2%PID_UPA_UPAR_PATHWAY	2.90E-02	1.89	3	20	Signaling
RESPONSE TO ZINC ION%GOBP%GO:0010043	2.90E-02	1.89	3	20	Membrane transport + ion balance
POSITIVE REGULATION OF STEROID METABOLIC PROCESS%GOBP%GO:0045940	2.90E-02	1.87	3	20	Metabolism
AMINO ACID TRANSPORT%GOBP%GO:0006865	2.96E-02	2.54	0	72	Membrane transport + ion balance
NEGATIVE REGULATION OF CELL-SUBSTRATE ADHESION%GOBP%GO:0010812	3.09E-02	1.85	1	44	Miscellaneous
NEUROTRANSMITTER METABOLIC PROCESS%GOBP%GO:0042133	3.15E-02	1.83	1	45	Neurotransmitter/synaptic regulation
REGULATION OF BEHAVIOR%GOBP%GO:0050795	3.15E-02	1.73	1	45	IR
GLYCOSPHINGOLIPID METABOLIC PROCESS%GOBP%GO:0006687	3.23E-02	1.87	2	34	Metabolism (lipid)
REGULATION OF ANION TRANSPORT%GOBP%GO:0044070	3.27E-02	2.02	0	76	Membrane transport + ion balance
DRUG TRANSPORT%GOBP%GO:0015893	3.37E-02	2.22	0	78	Membrane transport + ion balance
OTHER INTERLEUKIN SIGNALING%REACTOME DATABASE ID RELEASE 71%449836	3.58E-02	1.86	5	16	Signaling
BIOCARTA_EDG1_PATHWAY%MSIGDB_C2%BIOCARTA_EDG1_PATHWAY	3.69E-02	1.88	4	24	Signaling
OLIGOSACCHARIDE METABOLIC PROCESS%GOBP%GO:0009311	3.81E-02	1.74	4	25	Metabolism
PLASMA LIPOPROTEIN PARTICLE ORGANIZATION%GOBP%GO:0071827	3.81E-02	1.89	5	18	Miscellaneous
GLIAL CELL DEVELOPMENT%GOBP%GO:0021782	3.83E-02	1.84	0	86	Gliogenesis
REGULATION OF CELL-MATRIX ADHESION%GOBP%GO:0001952	3.83E-02	1.76	0	86	Extracellular matrix
ACYLGLYCEROL METABOLIC PROCESS%GOBP%GO:0006639	3.87E-02	1.82	1	55	Metabolism (lipid)
ADENYLATE CYCLASE-MODULATING G PROTEIN-COUPLED RECEPTOR SIGNALING PATHWAY%GOBP%GO:0007188	3.87E-02	1.71	0	83	Signaling
SIGNALING BY NTRK2 (TRKB)%REACTOME%R-HSA-9006115.2	3.96E-02	1.85	5	21	Signaling
RESPONSE TO MECHANICAL STIMULUS%GOBP%GO:0009612	4.36E-02	1.53	0	91	IR
MYELINATION%GOBP%GO:0042552	4.42E-02	1.98	0	94	Gliogenesis
MULTICELLULAR ORGANISMAL SIGNALING%GOBP%GO:0035637	4.43E-02	1.63	1	66	Signaling
UNSATURATED FATTY ACID METABOLIC PROCESS%GOBP%GO:0033559	4.51E-02	1.81	4	33	Metabolism (lipid)
MEMBRANE ASSEMBLY%GOBP%GO:0071709	4.58E-02	1.88	5	28	Miscellaneous
RESPONSE TO AMINO ACID%GOBP%GO:0043200	4.58E-02	1.68	1	68	Miscellaneous
ANION TRANSMEMBRANE TRANSPORT%GOBP%GO:0098656	4.71E-02	2.01	0	97	Membrane transport + ion balance
SYNAPSE ASSEMBLY%GOBP%GO:0007416	4.71E-02	1.66	1	70	Neurotransmitter/synaptic regulation
ION CHANNEL TRANSPORT%REACTOME DATABASE ID RELEASE 71%983712	4.78E-02	1.65	0	96	Membrane transport + ion balance
REGULATION OF CELL JUNCTION ASSEMBLY%GOBP%GO:1901888	4.94E-02	1.71	1	73	Miscellaneous
RESPONSE TO CADMIUM ION%GOBP%GO:0046686	4.96E-02	1.86	6	27	Membrane transport + ion balance
RECEPTOR-MEDIATED ENDOCYTOSIS%GOBP%GO:0006898	4.96E-02	1.82	0	101	Membrane transport + ion balance
GPCRS, OTHER%WIKIPATHWAYS_20191210%WP41%MUS MUSCULUS	4.96E-02	1.79	6	27	Signaling

Shown are gene sets that are positively correlated with time (increasing in the transition from embryonic RPs to adult NSCs) where FDR < 0.05, analyzed from the combined SGZ RP/NSC dataset (a total of 885 cells, cells highlighted in blue in Fig. 3*C*). Also shown are the adjusted *p* values (adj. *p* value; FDR), enrichment scores (Norm. Enr. score), the size of the gene set and the number of times a random gene set had a more extreme enrichment score than the gene set (nMoreExtreme). Gene sets are ordered from most to least significant from top to bottom. These gene sets were also categorized with regard to a number of broad categories, including neurotransmitter transport and synaptic regulation (neurotransmitter/synaptic regulation), ion regulation and membrane transport (ion balance + membrane transport), signaling pathways (signaling), gliogenesis and metabolism and lipid oxidation (metabolism). IR indicates they were not considered relevant to the NSCs and miscellaneous includes gene sets that do not fit into these categories.

### Upon activation, adult SGZ NSCs reacquire a development-like state that includes re-expression of proneurogenic genes

**Figure 7.**
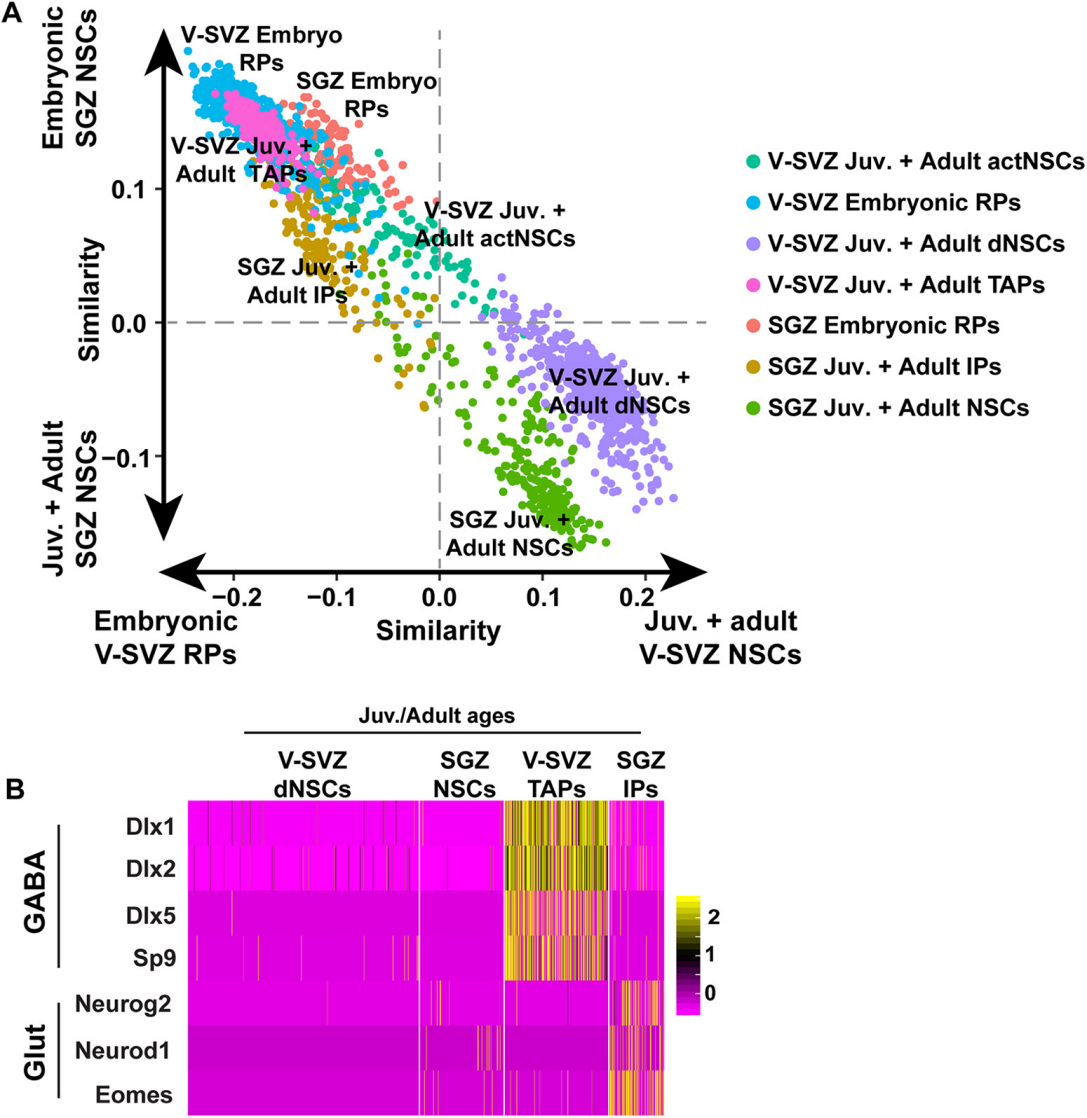
Upon activation, adult SGZ NSCs reacquire a development-like state that includes re-expression of proneurogenic genes. ***A***, Raw transcriptomes from the V-SVZ and SGZ NSC dataset shown in Figure 3*B* were merged with V-SVZ activated NSCs (actNSCs) and V-SVZ TAPs from juvenile and adult ages (P20/P34/P61; as defined in Borrett et al., 2020) as well as SGZ IPs from juvenile and adult ages (P18/P19/P23/P120/P132; as shown in Fig. 1*A*). Scatterplot shows single-cell correlation analysis of different V-SVZ and SGZ populations (color coded by cell type and age as defined in Fig. 3*A*), where individual transcriptomes were each correlated with averaged gene expression for E14 V-SVZ RPs versus juvenile/adult V-SVZ dormant NSCs (P20/P34/P61; *x*-axis), and with averaged gene expression for E16.5 SGZ RPs versus juvenile/adult SGZ NSCs (P18/P19/P23/P120/P132; *y*-axis). Gene expression values are not batch-corrected. ***B***, Single-cell heatmap illustrating expression of genes involved in GABAergic and glutamatergic neurogenesis in the juvenile and adult SGZ and V-SVZ precursor populations shown in panel ***A***. Genes are color-coded for levels of expression as per the adjacent color keys. Gene expression values are not batch-corrected.

Previous work (Borrett et al., 2020) showed that adult V-SVZ transit-amplifying cells (TAPs) exhibited an embryonic RP-like transcriptional program, implying that adult dormant NSCs reverted to an earlier developmental state when activated for cell genesis. To ask whether this was also true for adult SGZ NSCs, we performed a single-cell correlation analysis comparing dormant NSCs and their downstream activated NSC and TAP/IP progeny from the V-SVZ and SGZ (Fig. 7*A*). To perform this analysis, we determined average gene expression for E16.5 nonproliferative dentate gyrus RPs and juvenile/adult SGZ NSCs (P18–P132) as a first comparator (Fig. 7*A*, *y*-axis). As a second comparator, we determined average gene expression for E14 V-SVZ RPs and juvenile/adult V-SVZ dormant NSCs (P20/34/61; Fig. 7*A*, *x*-axis). We then correlated these average transcriptomes with single-cell transcriptomes from the E16.5 dentate gyrus RPs, adult SGZ NSCs and adult SGZ IPs. To enable a direct comparison, we also correlated single-cell transcriptomes from the V-SVZ dataset, including E14 cortical and GE RPs, adult dormant NSCs, adult activated NSCs and adult TAPs (all as defined in Borrett et al., 2020). This analysis showed that the various RP populations were largely but not completely intermingled, confirming that they were very similar to each other. Moreover, as previously published (Borrett et al., 2020), the adult V-SVZ TAPs were closely intermingled with the cortical and GE RPs. Notably, the adult SGZ IPs were closely mingled with the adult V-SVZ activated NSCs and were more highly correlated to embryonic RPs than to adult SGZ or V-SVZ dormant NSCs.

These data suggest that dormant SGZ and V-SVZ adult NSCs reacquire a development-like state when activated. We asked whether this was also true with regard to neurogenesis by examining genes associated with GABAergic (*Dlx1*, *Dlx2*, *Dlx5*, *Sp9)* and glutamatergic (*Neurog2*, *Neurod1*, *Eomes*) neurogenesis. We analyzed expression of these proneurogenic mRNAs in adult dormant SGZ and V-SVZ NSCs and in their TAP progeny, TAPs and IPs (the same adult transcriptomes included in Fig. 7*A*; V-SVZ activated NSCs at juvenile and adult ages were not included in this analysis). This analysis, shown as a single-cell heatmap (Fig. 7*B*) demonstrated that the proneurogenic mRNAs were detectably expressed in few of the dormant NSCs. However, many of the V-SVZ TAPs detectably expressed the GABAergic but not glutamatergic mRNAs, while many of the SGZ IPs expressed the glutamatergic but not GABAergic mRNAs. Thus, dormant postnatal V-SVZ and SGZ NSCs are not apparently transcriptionally primed for generating specific types of neurons. Instead, this proneurogenic priming, which is also observed in embryonic RPs (Fig. 5*B–D*), apparently only occurs in their downstream activated progeny.

### Identification of shared genes that are selectively increased in dormant adult NSCs

**Figure 8.**
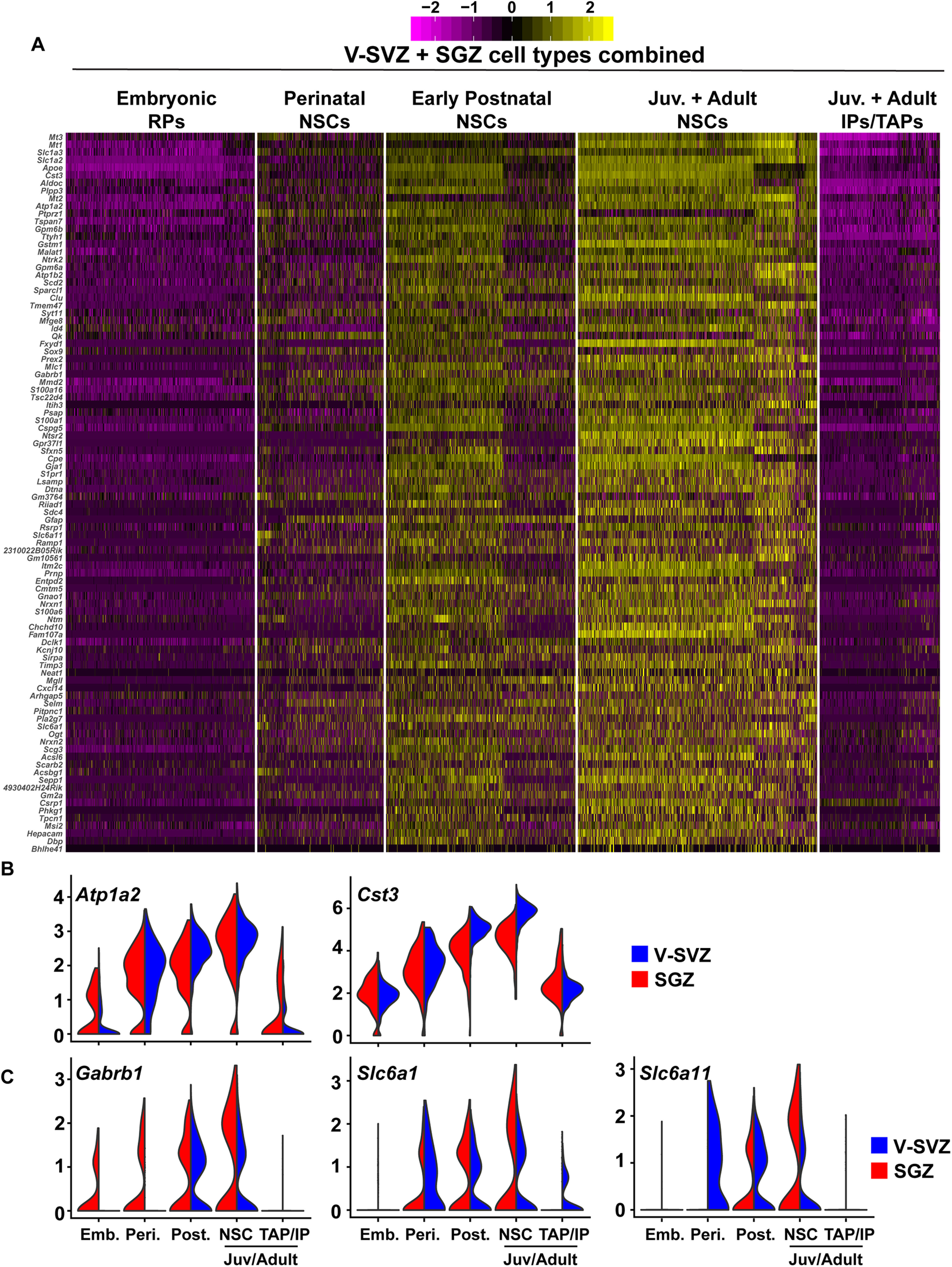
Identification of shared genes selectively enriched in dormant adult NSCs. ***A***, Single-cell heatmap showing the expression profiles of 94 genes selectively enriched in juvenile/adult V-SVZ and SGZ NSCs relative to embryonic NSCs and juvenile/adult V-SVZ transit-amplifying cells (TAPs) and SGZ IPs (same dataset as in Fig. 7*A* without the activated juvenile/adult V-SVZ NSCs). Each column line represents the level of expression in a single cell. Gene expression represents scaled expression and is color-coded as per the adjacent color key, where pink/purple represents no or low expression, and yellow the highest expression. Gene expression values are not batch-corrected. ***B***, Violin plots showing gene expression profiles of two selected mRNAs from ***A***, *Atp1a2* and *Cst3*, in the same populations as shown in ***A***. SGZ expression profiles are shown in red and V-SVZ profiles in blue. Gene expression values are not batch-corrected. ***C***, Violin plots showing gene expression profiles of three selected mRNAs from ***A***, *Gabrb1*, *Slc6a1*, and *Slc6a11*, all of which are involved with NSC responsiveness to GABA. Gene expression values are not batch-corrected.

These findings support a model where V-SVZ and SGZ precursors share many commonalities with regard to their transcriptional identity, developmental progression to dormancy and subsequent activation to make adult-born progeny. To further define their shared adult transcriptional state, we analyzed SGZ NSCs for mRNAs that were upregulated developmentally from embryogenesis to adulthood but then downregulated in activated adult IPs (Table 6). Notably, of 105 SGZ NSC mRNAs that fulfilled these criteria, 94 (90%) were also identified in a previous similar analysis of V-SVZ NSCs (Borrett et al., 2020). A single-cell heatmap confirmed that all 94 mRNAs were upregulated in V-SVZ and SGZ NSCs as they transitioned to dormancy postnatally and were then downregulated in the activated TAPs/IPs (Fig. 8*A*). Many of these genes were involved in sensing and responding to the adult niche environment, including genes for transport and buffering of neurotransmitters and ions, and for cell:cell and cell:extracellular matrix interactions (see Table 7 for functional annotations). They also included genes important for protecting these long-lived cells from adverse environmental events, such as genes involved in detoxification and lysosome function, as well as many genes involved in lipid metabolism. Examples include mRNAs encoding the sodium-potassium ATPase subunit *Atp1a2* and the secreted inhibitor of cysteine proteases *Cst3* (Fig. 8*B*). Notably, three of the 94 mRNAs encode proteins that functionally interact with the GABA neurotransmitter. These include the two GABA transporter mRNAs *Slc6a11* and *Slc6a1* and the GABA-A receptor subunit mRNA *Gabrb1* (Fig. 8*C*). These findings reinforce the idea that adult dormant NSCs are specialized for sensing and regulating their niche environments, and suggest that NSCs of both origins may alter their responses to GABA as they progress to a dormant state.

**Table 6:** Identification of a shared NSC gene signature enriched in juvenile/adult SGZ NSCs relative to embryonic SGZ RPs and juvenile/adult SGZ IPs (related to Fig. 8-10)

	Genes upregulated in juvenile/adult SGZ NSCs			
	vs E16.5 DG RPs		vs juvenile/adult SGZ IPs	
Gene	Average logFC	Adjusted *p* value	Average log FC	Adjusted *p* value
2310022B05Rik*	1.01	2.63E-04	1.12	7.80E-10
4930402H24Rik*	0.91	2.40E-02	0.86	2.70E-03
Acsbg1*	0.94	4.70E-03	0.88	1.66E-03
Acsl6*	0.92	5.81E-03	0.88	1.40E-03
Aldoc*	0.79	4.73E-03	2.64	1.17E-34
Ank2	1.30	9.97E-08	0.89	2.89E-04
Apoe*	2.16	3.11E-37	1.84	8.67E-40
Appl2	1.29	8.71E-08	1.07	6.96E-08
Arhgap5*	0.88	3.88E-02	0.85	2.19E-05
Atp1a2*	1.97	1.84E-30	1.75	3.13E-33
Atp1b2*	1.66	1.29E-17	1.72	3.98E-24
Bhlhe41*	1.05	4.41E-03	0.87	4.46E-02
Chchd10*	1.20	4.37E-06	1.05	4.14E-07
Clu*	1.17	6.96E-09	1.80	1.88E-23
Cmtm5*	1.35	5.95E-12	1.04	6.21E-09
Cpe*	1.65	6.97E-19	1.14	1.68E-13
Cspg5*	0.95	5.26E-03	1.39	1.87E-14
Csrp1*	1.09	1.64E-04	0.86	5.50E-03
Cst3*	2.53	9.43E-41	1.77	2.06E-39
Cxcl14*	1.03	3.12E-02	1.05	1.87E-05
Dbp*	0.90	5.80E-03	0.79	1.99E-02
Dclk1*	1.27	1.96E-10	0.93	7.08E-07
Dtna*	1.47	2.85E-11	1.26	3.46E-11
Entpd2*	0.93	4.31E-02	1.16	4.62E-09
Fam107a*	1.13	1.35E-06	1.05	4.22E-07
Fxyd1*	1.65	4.67E-14	1.56	9.34E-17
Gabrb1*	1.15	2.04E-05	1.46	1.34E-15
Gfap*	1.31	6.72E-07	1.24	3.09E-10
Gja1*	1.77	3.01E-13	1.45	2.59E-13
Gm10561*	1.70	1.98E-12	1.35	7.80E-10
Gm2a*	1.04	1.66E-02	0.88	2.74E-03
Gm3764*	0.58	1.26E-03	0.86	3.85E-11
Gnao1*	0.94	3.64E-04	1.03	7.22E-09
Gpm6a*	1.69	1.55E-23	1.48	4.40E-25
Gpm6b*	1.25	1.69E-23	1.34	1.41E-29
Gpr37l1*	1.65	9.83E-17	1.36	1.22E-13
Gria2	1.90	1.39E-23	0.99	1.39E-12
Gstm1*	1.83	8.38E-22	1.81	8.69E-29
Hepacam*	1.23	6.10E-07	0.88	1.90E-02
Hopx	1.38	3.68E-13	1.72	7.54E-24
Hopxos	0.74	3.39E-02	0.70	4.23E-02
Id4*	1.58	1.08E-10	1.64	4.10E-17
Itih3*	1.60	1.24E-13	1.48	8.70E-15
Itm2c*	0.98	1.98E-04	1.08	1.66E-09
Kcnj10*	1.16	1.21E-05	0.99	1.72E-06
Lgr4	1.20	1.86E-04	1.05	4.53E-03
Lsamp*	1.54	2.22E-12	1.30	6.65E-12
Malat1*	1.52	9.63E-33	1.12	2.07E-27
Mfge8*	0.97	7.49E-10	1.30	4.72E-20
Mgll*	1.02	1.52E-03	0.96	1.32E-05
Mlc1*	1.20	1.48E-06	1.42	9.39E-16
Mmd2*	0.75	3.13E-07	1.03	1.70E-15
Msi2*	1.31	7.15E-10	0.68	1.13E-02
Mt1*	2.48	1.36E-36	2.67	5.99E-45
Mt2*	1.65	2.14E-21	2.09	6.21E-34
Mt3*	1.68	1.84E-30	2.53	9.75E-46
Neat1*	1.29	1.82E-06	1.14	7.61E-06
Notch2	1.97	3.39E-09	1.74	2.44E-11
Nrxn1*	1.85	1.53E-20	0.98	7.26E-09
Nrxn2*	1.25	1.40E-07	0.89	3.74E-04
Ntm*	1.45	1.78E-08	1.21	1.58E-07
Ntrk2*	1.61	2.17E-21	1.63	5.14E-27
Ntsr2*	1.97	1.26E-18	1.51	4.64E-14
Ogt*	1.19	2.72E-06	0.83	3.69E-04
Padi2	1.41	2.91E-09	1.28	1.96E-09
Phkg1*	0.87	9.90E-03	0.82	5.55E-03
Pitpnc1*	1.01	8.58E-04	0.84	3.62E-05
Pla2g7*	1.03	1.53E-02	1.07	6.67E-05
Plpp3*	1.85	6.41E-30	1.71	4.37E-34
Prex2*	1.67	3.28E-16	1.41	1.60E-16
Prnp*	1.32	4.53E-09	1.08	2.60E-09
Psap*	1.22	2.34E-12	1.16	9.57E-15
Ptprz1*	1.55	9.55E-31	1.43	1.16E-32
Qk*	0.80	1.54E-06	1.08	8.16E-17
Ramp1*	1.27	3.03E-07	1.24	7.79E-10
Riiad1*	1.29	1.02E-04	1.39	2.04E-10
Rsrp1*	1.04	1.21E-06	1.03	3.30E-10
S100a1*	1.61	2.13E-15	1.32	1.38E-14
S100a16*	1.77	3.24E-18	1.34	2.48E-15
S100a6*	1.20	3.61E-06	1.16	3.61E-08
S1pr1*	1.33	3.43E-07	1.36	1.67E-12
Scarb2*	0.96	3.92E-03	0.78	1.59E-03
Scd2*	1.36	3.83E-20	1.35	4.04E-24
Scg3*	0.92	9.73E-03	0.80	7.60E-04
Sdc4*	1.49	8.05E-07	1.43	2.84E-10
Selm*	1.02	1.12E-03	0.85	2.95E-05
Sepp1*	1.05	2.97E-05	0.77	1.99E-03
Sfxn5*	2.13	1.16E-14	1.70	1.27E-13
Sirpa*	1.05	1.84E-03	1.02	2.00E-06
Slc14a1	0.90	3.74E-04	0.89	1.47E-05
Slc1a2*	3.39	1.71E-40	2.22	3.72E-41
Slc1a3*	1.92	2.94E-40	1.80	1.38E-44
Slc25a18	1.36	4.21E-10	1.23	3.79E-11
Slc6a1*	1.34	3.20E-06	1.08	2.00E-04
Slc6a11*	1.50	1.40E-12	1.21	4.39E-10
Sox9*	0.99	7.59E-07	1.36	1.22E-16
Sparcl1*	2.17	9.21E-24	1.72	1.27E-23
St6galnac5	1.12	1.10E-05	1.03	6.65E-06
Syt11*	0.92	2.17E-12	1.18	2.88E-21
Timp3*	0.99	6.78E-03	0.98	4.02E-06
Tmem47*	1.62	3.77E-17	1.56	1.00E-21
Tpcn1*	0.87	2.01E-02	0.77	6.07E-03
Tsc22d4*	0.95	4.13E-05	1.30	3.73E-15
Tspan7*	1.69	6.08E-26	1.68	5.68E-31
Ttyh1*	1.09	3.38E-10	1.93	2.61E-29

**Figure 9.**
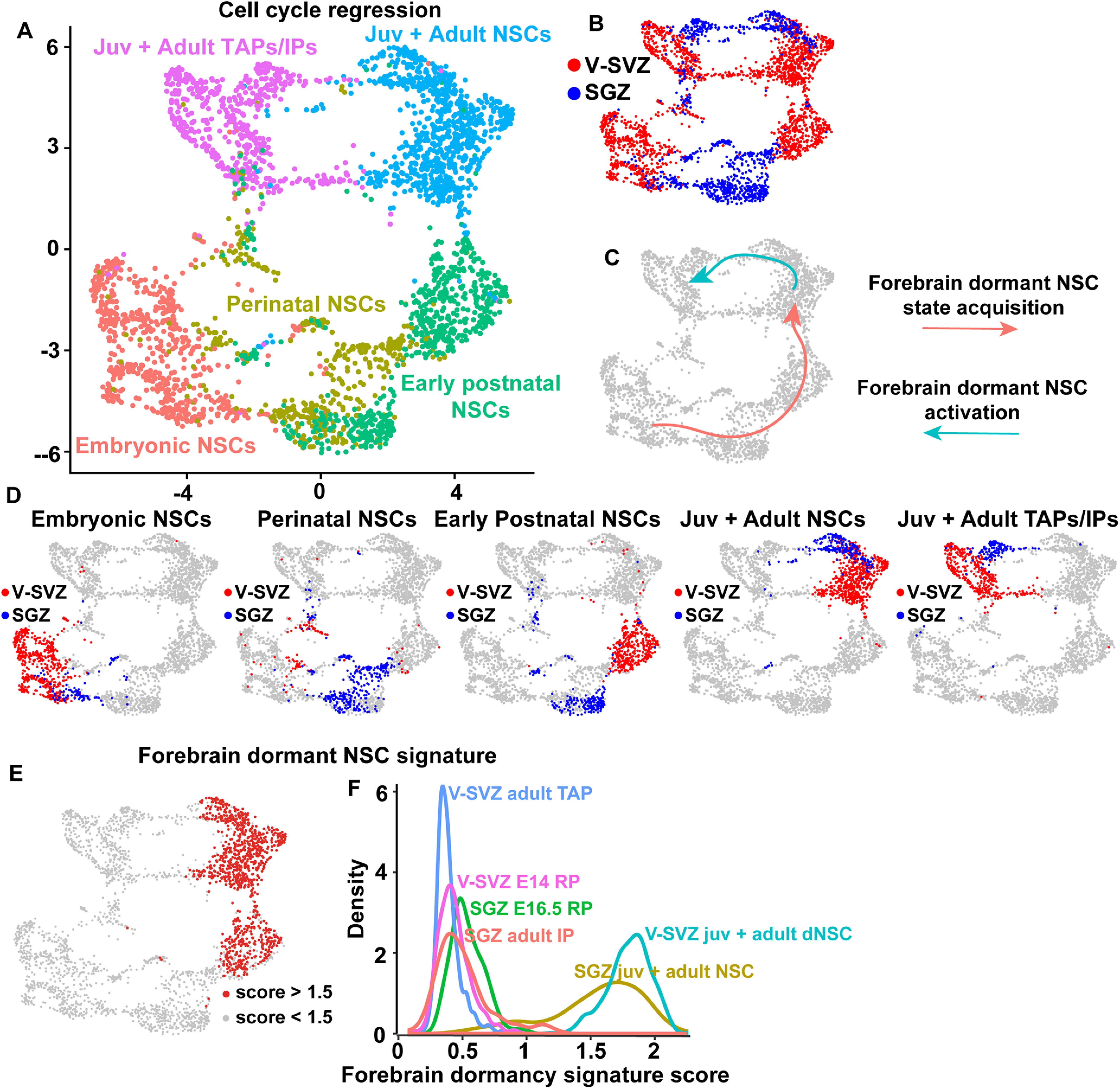
Identification of a shared adult dormant NSC gene signature. ***A***, Transcriptomes of embryonic, perinatal, early postnatal, juvenile and adult V-SVZ and SGZ NSCs were combined with those of juvenile and adult V-SVZ and SGZ TAPs/IPs (same dataset as shown in Fig. 7*A*), cell cycle genes were regressed, and the dataset was run through the batch-corrected pipeline. Shown is a UMAP visualization where cells are color-coded and labeled based on cell type. Note here that the Juv + Adult TAPs/IPs group shown in purple include V-SVZ activated NSCs at juvenile and adult ages. ***B***, UMAP plot as in ***A*** with cells color coded based on region of origin (V-SVZ or SGZ). ***C***, UMAP plot as in ***A*** annotated to depict two distinct trajectories. The first trajectory describes the progression from embryonic RP to adult NSC in the V-SVZ and SGZ (pink). The second trajectory describes the progression from dormant juvenile/adult NSCs to activated, differentiating TAPs/IPs (light blue). ***D***, UMAP visualizations as in ***A*** overlaid with V-SVZ and SGZ cell types from different ages, as defined in Figure 3*A*. V-SVZ cells are shown in red and SGZ cells in blue. ***E***, UMAP as in ***A*** overlaid with gene expression scores for a shared adult dormant NSC signature consisting of the 94 genes shown in the heatmap in Figure 8*A* and in Table 7. Red denotes cells with scores >1.5. ***F***, Density plot showing the distribution of the shared adult dormant NSC signature scores in V-SVZ juvenile/adult TAPs (P20/P34/P61; blue), SGZ juvenile/adult IPs (P18/P19/P23/P120/P132; orange), V-SVZ E14 RPs (pink), E16.5 SGZ RPs (green), V-SVZ juvenile/adult dormant NSCs (dNSCs; turquoise), and juvenile/adult SGZ NSCs (yellow).

**Figure 10.**
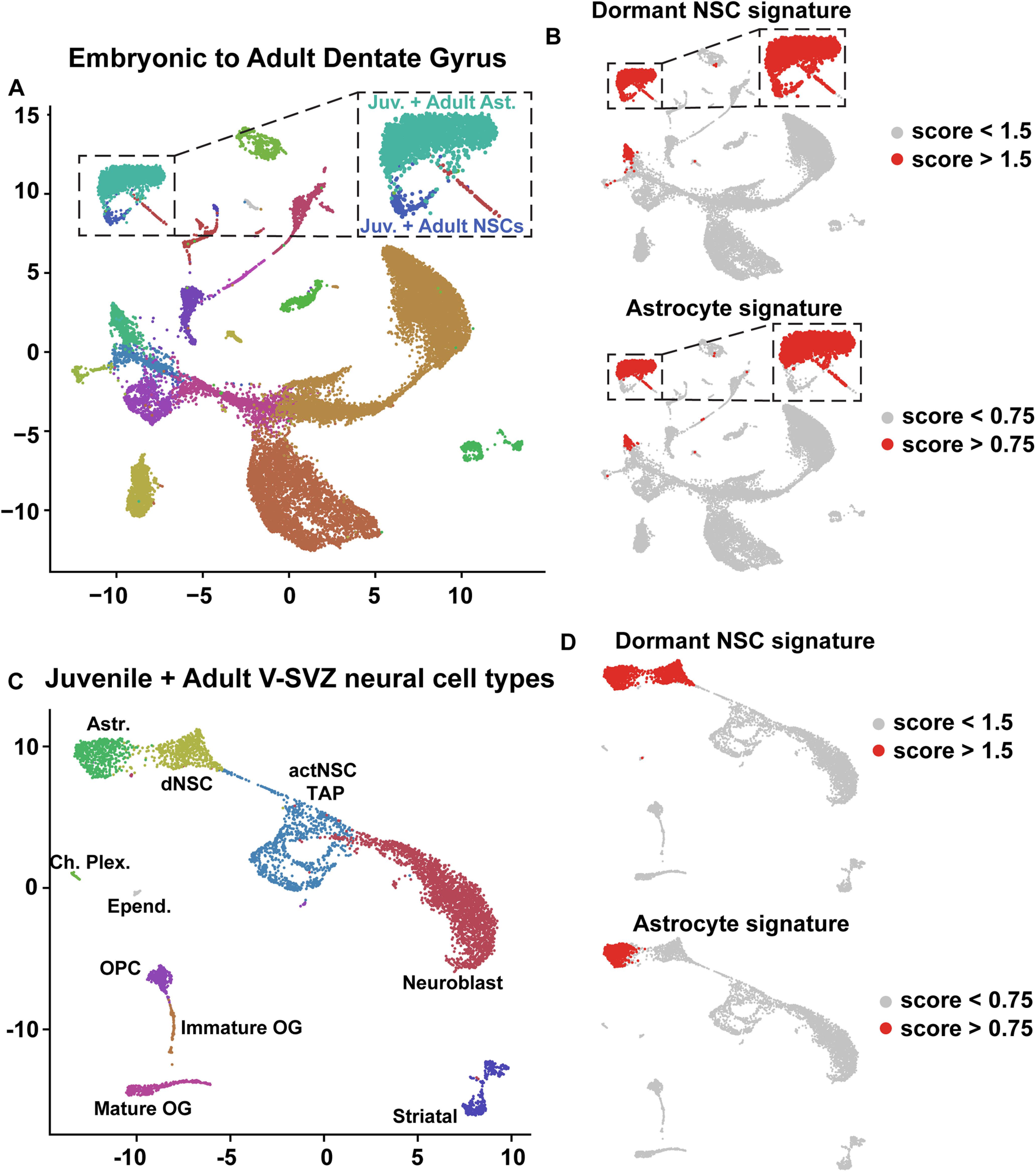
Analysis of the shared adult dormant NSC gene signature in all V-SVZ and SGZ cells. ***A***, ***B***, UMAP visualization of dentate gyrus cells as in Figure 1*A* overlaid with expression scores for two different gene signatures, the shared 94 gene dormant adult NSC signature (panel ***B***, top) and the shared 26 gene niche astrocyte signature (panel ***B***, bottom). The region shown in the hatched boxes includes juvenile/adult astrocytes and NSCs as identified in Figure 1*B* and is shown at a larger size to the right in each case. Red denotes cells with scores >1.5 (top) or 0.75 (bottom). Data are not batch-corrected. ***C***, ***D***, Annotated UMAP visualization of juvenile/adult V-SVZ neural cells (P20, P34, P61 combined) as shown in Figure 2*E*, overlaid with expression scores for two different gene signatures, the shared 94 gene dormant NSC signature (panel ***D***, top) and the shared 26 gene niche astrocyte signature (panel ***D***, bottom). Red denotes cells with scores >1.5 (top) or 0.75 (bottom). Data are not batch-corrected.

Differential gene expression was performed for (1) juvenile/adult SGZ NSCs (P18–P132; blue cells in two final right panels in Fig. 3*C*) versus E16.5 SGZ RPs (blue cells in left panel in Fig. 3*C*) and (2) juvenile/adult SGZ NSCs versus juvenile/adult SGZ IPs (P18–P132; 139 cells). This analysis identified 105 genes that were significantly enriched in juvenile/adult SGZ NSCs relative to both E16.5 SGZ RPs and juvenile/adult SGZ IPs (log fold change > 0.5, adj. *p* value < 0.05; FWER). These 105 genes are shown along with their fold change in expression and adjusted *p* values. Also indicated with an asterisk are 94 of these genes that were also enriched in juvenile/adult V-SVZ dormant NSCs relative to E14 V-SVZ RPs and juvenile/adult V-SVZ TAPs, as identified in Borrett et al. (2020). These 94 genes were used to define a shared adult dormant NSC gene signature. Analysis using this 94 gene signature is described in Figures 8-10.

We asked whether this group of differentially-enriched genes would specifically identify adult dormant NSCs. To do this, we combined transcriptomes for all V-SVZ and SGZ NSC populations with those of the adult IPs/TAPs, removed the cell cycle genes, and then ran them through the Harmony batch-corrected pipeline together. UMAP visualization identified four main groups of transcriptomes that were segregated by developmental stage and/or activation state; embryonic RPs, perinatal NSCs, juvenile/adult NSCs, and IPs/TAPs (Fig. 9*A–D*). Each group included cells of both V-SVZ and SGZ origin that were closely-associated, but only partially intermingled, consistent with the conclusion that they were very similar but not identical. We then used the 94 enriched dormant NSC genes (Table 7) to compute single-cell gene signature scores for these transcriptomes. This gene signature was very specific to the dormant NSCs from P6/7 through to adulthood (Fig. 9*E*,*F*).

**Table 7:** Categorization and expression of the shared adult dormant NSC signature genes in juvenile/adult V-SVZ and SGZ NSCs and astrocytes (related to Fig. 8-10)

	Gene abundance (%)				
	V-SVZ populations		SGZ populations		
Genes	V-SVZ dNSCs	V-SVZ Astr	SGZ NSCs	SGZ Astr	Category
2310022B05Rik	54.53	48.26	59.91	45.89	Miscellaneous
4930402H24Rik	54.19	54.18	33.02	41.24	Miscellaneous
Acsbg1	63.42	88.50	34.91	69.60	Metabolism (lipid)
Acsl6	60.00	87.46	26.42	69.07	Metabolism (lipid)
Aldoc	98.97	99.48	81.60	99.18	Metabolism
Apoe	99.83	100.00	98.58	100.00	Miscellaneous
Arhgap5	61.20	74.56	55.19	59.63	Signaling
Atp1a2	98.63	99.83	90.09	99.66	Ion + neurotransmitter regulation
Atp1b2	81.20	96.34	74.53	96.90	Ion + neurotransmitter regulation
Bhlhe41	12.14	17.94	21.23	18.83	Gene regulation + RNA binding
Chchd10	75.56	87.98	40.57	81.22	Mitochondrial gene
Clu	98.46	99.83	72.17	99.23	Miscellaneous
Cmtm5	65.30	79.09	47.17	40.17	Signaling
Cpe	100.00	99.30	82.55	98.55	Metabolism
Cspg5	99.49	97.91	59.43	89.74	ECM + adhesion
Csrp1	85.47	91.29	41.04	58.23	Gene regulation + RNA binding
Cst3	100.00	100.00	100.00	99.95	ECM + adhesion
Cxcl14	48.55	93.90	25.47	76.52	Miscellaneous
Dbp	47.18	56.79	28.77	33.54	Gene regulation + RNA binding
Dclk1	85.47	93.73	70.28	83.20	Signaling
Dtna	76.58	65.85	49.06	53.78	ECM + adhesion
Entpd2	38.29	51.92	32.55	19.80	Metabolism
Fam107a	87.18	90.07	31.60	72.99	Miscellaneous
Fxyd1	96.24	86.06	54.72	59.54	Ion + neurotransmitter regulation
Gabrb1	48.38	70.03	53.77	62.68	Ion + neurotransmitter regulation
Gfap	30.43	12.37	49.06	71.10	Miscellaneous
Gja1	92.65	98.43	60.85	99.81	ECM + adhesion
Gm10561	55.56	42.33	49.06	21.06	Miscellaneous
Gm2a	48.89	51.39	39.62	58.52	Miscellaneous
Gm3764	76.41	89.55	83.02	82.58	Miscellaneous
Gnao1	82.91	78.75	56.13	48.50	Signaling
Gpm6a	83.08	96.52	87.74	99.47	Signaling
Gpm6b	98.97	95.82	94.81	97.48	Signaling
Gpr37l1	81.03	99.13	55.66	90.42	Signaling
Gstm1	98.12	96.34	78.30	88.14	Detoxification
Hepacam	75.21	84.84	38.21	62.78	ECM + adhesion
Id4	93.16	93.38	60.38	67.09	Gene regulation + RNA binding
Itih3	26.50	30.49	49.06	23.62	ECM + adhesion
Itm2c	95.21	91.11	55.66	64.86	Miscellaneous
Kcnj10	28.38	22.13	50.47	51.50	Ion + neurotransmitter regulation
Lsamp	67.52	92.51	57.08	74.30	ECM + adhesion
Malat1	100.00	97.39	100.00	99.85	Miscellaneous
Mfge8	94.70	96.52	82.55	80.74	ECM + adhesion
Mgll	54.19	66.20	37.74	61.08	Metabolism (lipid)
Mlc1	91.97	87.63	59.43	79.57	Ion + neurotransmitter regulation
Mmd2	92.48	97.56	81.60	85.24	Signaling
Msi2	84.44	68.64	59.91	41.97	Gene regulation + RNA binding
Mt1	99.32	99.48	98.58	99.95	Detoxification
Mt2	97.78	98.26	94.81	97.97	Detoxification
Mt3	98.97	99.65	99.06	100.00	Detoxification
Neat1	26.67	23.69	34.91	44.68	Miscellaneous
Nrxn1	75.38	81.01	66.04	74.59	ECM + adhesion
Nrxn2	53.16	58.71	47.17	58.18	ECM + adhesion
Ntm	59.49	88.68	45.28	64.86	ECM + adhesion
Ntrk2	96.92	94.60	79.72	94.39	Signaling
Ntsr2	88.38	98.61	59.43	87.85	Signaling
Ogt	76.92	70.91	58.96	41.63	Metabolism
Phkg1	49.06	73.87	21.70	37.46	Signaling
Pitpnc1	56.24	49.13	47.64	36.59	Metabolism (lipid)
Pla2g7	43.76	86.24	33.96	73.23	Metabolism (lipid)
Plpp3	98.29	98.78	92.45	98.69	ECM + adhesion
Prex2	68.89	77.18	60.85	62.00	Signaling
Prnp	98.80	99.13	62.26	79.77	Miscellaneous
Psap	96.41	95.47	74.53	93.47	Miscellaneous
Ptprz1	85.13	93.38	96.70	94.29	Signaling
Qk	76.92	70.56	83.02	92.21	Gene regulation + RNA binding
Ramp1	57.26	75.09	41.98	43.76	Signaling
Riiad1	50.09	11.15	41.98	3.19	Signaling
Rsrp1	95.73	90.77	69.34	70.04	Miscellaneous
S100a1	86.84	80.14	60.85	71.10	Ion + neurotransmitter regulation
S100a16	88.38	88.50	66.51	62.44	Ion + neurotransmitter regulation
S100a6	85.47	46.86	41.04	29.33	Ion + neurotransmitter regulation
S1pr1	73.50	92.16	53.77	89.06	Signaling
Scarb2	30.60	27.00	41.04	36.98	Miscellaneous
Scd2	92.65	78.92	88.68	96.85	Metabolism (lipid)
Scg3	86.32	95.47	50.47	81.99	Miscellaneous
Sdc4	59.66	67.07	45.28	73.86	ECM + adhesion
Selm	66.32	44.60	54.25	42.55	Miscellaneous
Sepp1	80.68	95.64	33.96	84.27	Detoxification
Sfxn5	65.64	58.71	64.15	67.42	Ion + neurotransmitter regulation
Sirpa	60.17	46.34	34.91	47.53	Signaling
Slc1a2	99.32	99.48	96.23	100.00	Ion + neurotransmitter regulation
Slc1a3	98.12	99.13	99.06	99.56	Ion + neurotransmitter regulation
Slc6a1	48.72	74.04	44.81	77.44	Ion + neurotransmitter regulation
Slc6a11	35.21	62.89	47.64	76.86	Ion + neurotransmitter regulation
Sox9	75.21	64.81	73.58	76.23	Gene regulation + RNA binding
Sparcl1	76.75	99.65	71.70	97.10	ECM + adhesion
Syt11	85.64	73.87	85.85	60.99	Miscellaneous
Timp3	71.28	74.39	33.49	42.55	ECM + adhesion
Tmem47	77.61	83.28	75.00	87.22	ECM + adhesion
Tpcn1	39.32	30.14	30.19	17.91	Ion + neurotransmitter regulation
Tsc22d4	86.32	92.51	68.40	76.38	Gene regulation + RNA binding
Tspan7	97.78	99.13	89.62	96.42	ECM + adhesion
Ttyh1	91.28	97.21	77.83	97.14	Ion + neurotransmitter regulation

Shown are the 94 shared adult dormant NSC genes, and the relative proportions of juvenile/adult V-SVZ and SGZ NSCs and astrocytes that detectably express these genes. The astrocytes in this analysis included the green cells in Figures 1*B* (SGZ) and 2E (V-SVZ). The shared genes were also categorized with regard to a number of broad cellular processes including metabolism, cell signaling, ion and neurotransmitter regulation, cell adhesion and the extracellular matrix (ECM), gene regulation and RNA binding, and detoxification.

These gene enrichment studies provide insights into the common transcriptional ground-state of dormant postnatal NSCs. However, further analysis showed that, with the exception of *Riiad1*, all of the mRNAs in this enriched dataset were also expressed by niche astrocytes (Table 7). We therefore asked whether we could combine the dormant NSC signature with the astrocyte gene signature (Fig. 1*G*) to specifically identify dormant adult NSCs in the V-SVZ and SGZ. To do this, we overlaid both gene signatures on the complete dentate gyrus dataset (shown in Fig. 1*A*) and on the juvenile/adult V-SVZ neural cell dataset (shown in Fig. 2*E*), as visualized by UMAPs. As predicted, in both the SGZ and V-SVZ datasets the adult dormant NSCs were identified by the NSC but not the astrocyte gene signature, while the niche astrocytes were positive for both (Fig. 10*A–D*). These findings provide a way to definitively identify dormant adult NSCs in the V-SVZ and SGZ from other niche cell types and reinforce the conclusion that while adult dormant NSCs and niche astrocytes are very similar they can be distinguished transcriptionally.

## Discussion

Analyses presented here provide insights into the identity and genesis of the two best-characterized NSC populations in the mammalian brain, forebrain V-SVZ NSCs that generate inhibitory OB interneurons and hippocampal SGZ NSCs that make excitatory dentate granule neurons. Our analyses support the conclusion that while these two NSC populations are not transcriptionally identical to each other, they are nonetheless very similar and share a common dormant adult NSC transcriptional ground state. Moreover, the transcriptional similarities between these two populations are seen throughout their lifespans, commencing when they are embryonic RP populations residing in adjacent regions around the lateral ventricle and being maintained as they progress over an extended postnatal period to become dormant adult NSCs. These findings are particularly important in light of previous work showing that transplantation of embryonic or postnatal NSCs from one niche to the other or one time point to the other is apparently sufficient for them to start making cells appropriate to their new environment (Fishell, 1995; Suhonen et al., 1996; Hitoshi et al., 2002; Sequerra et al., 2010). Our own computational analyses together with these previous transplant studies provide support for a model where V-SVZ and SGZ NSCs share a common ground state and where the cellular progeny they generate may be largely determined by their niche environment. While this model requires further experimental validation, it has important implications for attempts to regulate and environmentally reprogram endogenous cell genesis as a therapeutic strategy.

One of the key findings described here involves the NSC transition into and out of a dormant adult state. With regard to the developmental transition to dormancy, our analyses here build on previous work by Borrett et al. (2020) and demonstrate that V-SVZ and SGZ NSCs share a similar, temporally aligned trajectory of transcriptional shut-down. In the V-SVZ this transition to dormancy is a prolonged process that commences during late embryogenesis and extends into the third postnatal week, with the early postnatal NSCs displaying an intermediary transcriptional state (Borrett et al., 2020). Our analyses here indicate that the transition occurs over a similar timeframe in the SGZ, with early postnatal hippocampal NSCs in a transition state, and near complete acquisition of the adult dormant state occurring by the third postnatal week. What then is the dormant forebrain NSC state? For adult V-SVZ and SGZ NSCs, this dormancy state predominantly involves a downregulation of basic cellular processes such as those required for DNA replication and transcription, RNA processing and translation, ribosome biogenesis, and protein synthesis and folding, in good agreement with what has been described in other studies (Llorens-Bobadilla et al., 2015; Shin et al., 2015; Dulken et al., 2017; Berg et al., 2019; Xie et al., 2020). However, the dormant NSC state involves more than just this shut-down. The gene set enrichment analyses presented here and in Borrett et al. (2020) show that it also includes upregulation of transcriptional programs involved in sensing the niche environment, including membrane transport, ion balance regulation, neurotransmitter regulation, and cell surface receptor signaling. Intriguingly, our comparison of adult dormant NSCs with TAP/IPs demonstrated that many of these same genes are turned-off again when dormant NSCs are reactivated to generate their adult-born progeny. Intriguingly, at least some of these genes and processes are important for the maintenance of adult quiescent-like NSCs (Zhou et al., 2018; Obernier and Alvarez-Buylla, 2019; Kjell et al., 2020). Thus, dormancy is normally thought of as a “silent” stem cell state, but our analyses suggest that while adult NSCs are metabolically quiet, they are nonetheless actively monitoring and responding to their niche environments as previously suggested (Shin et al., 2015).

Our studies emphasize commonalities between SGZ and V-SVZ NSCs, but these are clearly distinct stem cell populations that make different types of neurons. Do our analyses provide insights into this differential neurogenesis? In the postnatal brain, V-SVZ NSCs make GABAergic interneurons, but they derive, in part, from cortical RPs that make excitatory glutamatergic neurons during embrogenesis (Kohwi et al., 2007; Ventura and Goldman, 2007; Fuentealba et al., 2015; Borrett et al., 2020; Zhang et al., 2020). These cortical RPs are located immediately adjacent to the dentate neuroepithelial RP parents of SGZ NSCs that make excitatory granule neurons. By contrast, most V-SVZ NSCs derive from subpallial GE RPs that make GABAergic neurons throughout life. Somewhat surprisingly, despite these differences in neurogenesis, our analyses, together with those previously published in Borrett et al. (2020), indicate that all three embryonic RP populations are very similar. Nonetheless, they are not identical, and both cortical and dentate neuroepithelial RPs are highly enriched for a small group of genes important for their pallial identity and embryonic excitatory neurogenesis. Conversely, the GE RPs are instead enriched for genes that are associated, in part, with a subpallial identity and GABAergic neurogenesis. Thus, a small cohort of genes is apparently sufficient to drive functional differences in embryonic neurogenesis.

Data presented here indicate, however, that the situation is different in the postnatal brain. Specifically, data presented here and in Borrett et al. (2020) show that postnatal dormant V-SVZ and SGZ NSCs do maintain a transcriptional memory of their embryonic origin, but also show that they do not detectably express proneurogenic genes. Instead, these genes become re-expressed when dormant NSCs are reactivated. Thus, while embryonic RPs are transcriptionally-primed to make the appropriate types of neurons (for example, see Zahr et al., 2018), dormant postnatal NSCs are apparently in an unbiased transcriptional state. A key question, then, is whether this means that postnatal NSCs are malleable with regard to the types of neurons they can generate. This possibility is suggested by the aforementioned transplant studies (Suhonen et al., 1996; Sequerra et al., 2010), by a number of developmental studies showing flexibility in GABAergic versus glutamatergic neurogenesis in embryonic forebrain precursors depending on their local environment (Machon et al., 2005; Willaime-Morawek et al., 2006; Azim et al., 2014; Zhang et al., 2020), and by previous work demonstrating adult genesis of neurons other than GABAergic OB neurons and dentate gyrus granule cells following injury (Magavi et al., 2000; Nakatomi et al., 2002; Chen et al., 2004; Brill et al., 2009). However, it is also possible that dormant NSCs maintain a neurogenic memory at the chromatin level and that, like many other facets of their cell biology, this transcription is silenced during dormancy. Definitively distinguishing these alternatives will require further experimentation.

Our analyses also indicate that, as seen for V-SVZ NSCs (Borrett et al., 2020), SGZ NSCs acquire a global development-like transcriptional state when they are reactivated to make adult-born neurons. In both cases the transition from a dormant to an active NSC involves an increase in metabolic genes/processes associated with an active, ultimately proliferative cell state, induction of gene sets associated with translation and adult cell genesis, and a coincident transcriptional shut-down of dormancy-associated genes. This recapitulation of a developmental state supports the idea that embryonic RPs and adult NSCs may be similar cells that are simply in different states of activation. Notably, one prediction of this model is that cues known to regulate embryonic RPs might have the same effect on adult NSCs, although this relatively straightforward prediction is somewhat complicated by the fact that niche environments differ and signaling is context-dependent.

One final conclusion involves the transcriptional commonalities between adult dormant NSCs and niche astrocytes. Analyses here and in Borrett et al. (2020) show that these two cell types can be readily distinguished on a transcriptional level. However, almost all of the genes enriched in adult dormant NSCs relative to developing and reactivated precursors were also enriched in astrocytes. What is the explanation for this latter finding? One previously-described hypothesis is that astrocytes may possess latent precursor-like properties. In support of this concept, it has previously been shown that parenchymal astrocytes can acquire a neurogenic potential following genetic or environmental alterations. For example, astrocytes can be reprogrammed to make neurons following overexpression of neuronal specifiers such as *NeuroD*, *Ascl1*, and *Neurog2* (Guo et al., 2014; Liu et al., 2015; Gascón et al., 2016). Moreover, blocking Notch signaling in parenchymal astrocytes following cortical injury is sufficient to induce a neurogenic program that resembles V-SVZ neurogenesis (Zamboni et al., 2020). A second hypothesis comes from our observation that most of the shared astrocyte/dormant NSC genes are involved in cell adhesion, the extracellular matrix, and ion and neurotransmitter sensing and regulation. Thus, we posit that perhaps astrocytes and dormant NSCs share a requirement for adhering within their niches, and then sensing, detoxifying and responding to those environments in unique ways. Perhaps, as has been suggested for astrocytes in the gray matter (Freeman and Rowitch, 2013), dormant NSCs must act to ensure that the V-SVZ and SGZ niches are favorable environments for their newborn neuroblast progeny. While this is not a function normally ascribed to NSCs, it might in part explain the degradation of these two niches that occurs when NSCs become depleted during aging (Conover and Shook, 2011).

## Synthesis

Reviewing Editor: Benjamin Deneen, Baylor College of Medicine Decisions are customarily a result of the Reviewing Editor and the peer reviewers coming together and discussing their recommendations until a consensus is reached. When revisions are invited, a fact-based synthesis statement explaining their decision and outlining what is needed to prepare a revision will be listed below. The following reviewer(s) agreed to reveal their identity: Ola Hermanson.

The goal of this study is define differences in the molecular profiles of adult NSC populations from the two predominant NSC niches in the brain: SGZ of the hippocampus and SVZ of the lateral ventricle. Mining previously published single cell data sets from NSC populations from these distinct niches, the authors perform a series of computational analyses between these data sets, identifying many similarities between these populations. The conclusions reached by the authors is that despite producing phenotypically distinct neuronal populations, these two NSC populations are transcriptionally similar and that these differences in cell fate production are likely the result of the surrounding microenvironment.

Overall, the reviewers felt that the conclusions reached by the authors were justified and would be an important contribution to the emerging field of cellular diversity in the adult brain. Despite these strengths, there were a few noted weaknesses that need to be addressed before this paper is suitable for publication. Below is a summary of the key points raised by the reviewers that can be addressed in a revised manuscript.

1) Use of embryonic radial glia data sets as a point of comparison with SGZ and SVZ profiling data needs further rationale

2) Harmony based batch corrections need additional biological rationale.

3) Authors need to account for distinct neuronal subtypes generated by SVZ (inhibitory-GABergiA) and SGZ (excitatory-Glutamatergic). Also is there evidence for NSC responses to neurotransmitters, particularly in the SGZ, which seems to regulated by GABA signaling

4) Validation of the computational findings is lacking

5) Comment on the comparision with Borett, et al in the Discussion

6) explanatory sentence in association with the analysis summarising the aim of using a particular transcript for the analysis for a non-single-cell-bioinformatic person.

7) explanatory sentences in association with the analysis that summarizes the logic underlying the use of a particular transcript for the analysis for a non-single-cell-bioinformatic person.

Reviewer comments

Reviewer 1

In this manuscript, the authors pursue extensive bioinformatic analyses based on in principle two published papers, Borrett et al., Cell Rep, 2020, and Hochgerner et al., Nat Neuro, 2018. The analyses unveil previously poorly understood differences and similarities between anatomically and/or temporally distinct neural stem cell (NSC) populations. The manuscript is well-written, timely, conclusions are sound and the discussion relevant, and in addition to the novel findings, the datasets can be used by other authors in the field, for example those interested in the roles for specific genes in various NSC populations. The manuscript also paves the way for future studies of how ECM factors play a role for the NSC state and fate. I have only minor remarks.

1. In the Borrett et al. paper, the similarity between GE and cortical NSCs are highlighted. After re-reading that paper, there are indeed some differences pointed out there, and these are also pointed out here, but in the present manuscript, the similarities between cortical and early SGZ NSCs are now highlighted, and the GE NSCs are described almost like a different population. The number of genes common and analysed are displayed, but for the sake of clarity, this reviewer would very much appreciate a paragraph, in the Discussion or integrated in the Results, clarifying how the authors look at this conceptually when compared to the conclusions of the Borrett et al paper. Looking for example at figure 3E, it almost goes against the Borrett paper. If I have misunderstood, please explain.

2. The SGZ NSCs in particular are very much linked to GABA signaling, and the response as we know differs from the early NSCs that responds with a depolarization whereas later the granule cells of course respond with a hyperpolarization. Is there anything in the gene expression data that would suggest differences in GABA responses between the (anatomically or temporally distinct) populations of NSCs or is this similar?

3. A problem with the single cell gene expression field is the vast number of analysis transcripts, and it is a bit hard for the reader to follow, also in this manuscript (Harmony is pretty new, "our pipeline" etc). It would be very much appreciated if there could be an explanatory sentence in association with the analysis summarising the aim of using a particular transcript for the analysis for a non-single-cell-bioinformatic person. In some cases (e.g., second paragraph, page 12), the authors do this very well, but in other the reader must read or re-read the original publications to understand the purpose.

4. Very minor: While some abbreviations are explained multiple times (e.g., GSEA), others are only in the abstract, please add such to the main text. Liu et al in the references are not placed in right order.

Reviewer 2

In this manuscript, the authors take advantage of previously generated scRNA sequencing datasets from several sources, to temporally examine the transcriptional states of dentate and SVZ NSCs from embryogenesis to adulthood. The goal of the study is to define dentate vs. SVZ NSC differences at the transcriptional level contributing to the production of excitatory dentate granule neurons vs. inhibitory olfactory bulb interneurons, respectively. Using scRNA sequencing analysis pipeline, the authors used cortical radial glial progenitor gene expression profile as a template, and compared dentate vs. SVZ NSCs from different developmental time points to adulthood. The pipeline included batch correction and correlation analyses, and found shared genes selectively enriched in dormant adult NSCs, and analyzed these NSC gene signatures. The authors concluded that even though dentate and SVZ NSCs generate distinct neuronal progeny, these two NSC populations are transcriptionally similar and that their stem cell niche environments may determine progeny identity.

While the figures generated from the scRNA seq analyses looked impressive, this manuscript is based purely on previously generated and published scRNA sequencing datasets (Hochgerner et al. 2018; Borrett et al. 2020). This manuscript also lacked biological validation of the outputs from the computational work. With the stated goal to define transcriptional level differences contributing to progeny fate differences between adult dentate and SVZ NSCs, I have some significant concerns regarding the experimental design and approach.

1) To compare transcriptional programs between dentate and SVZ NSCs, the authors began the analysis by examining 79 genes previously shown to be highly enriched in embryonic cortical radial glial progenitors (Yuzwa et al. 2017). It has been well documented that embryonic cortical radial glial progenitors represent a diverse cellular population. Thus, it is curious that the authors decided to select 79 genes that have been shown to be commonly enriched in cortical radial glial progenitors as a starting point for analyzing dentate and SVZ postnatal and adult NSCs. It biases the transcriptional signatures to represent only those that are common between diverse neural progenitors (generalizing neurogenic and gliogenic activities). The rationale for this approach is unclear and does not appear meaningful.

2) I find the Harmony-inspired batch correction and resulting downstream analyses highly problematic (Supp Fig. 2). To implement this batch correction between dentate and SVZ NSCs, the authors assumed that vascular endothelial cells from the adult dentate and SVZ should have identical transcriptional signatures. However, there is no evidence that vascular endothelial cells from distant brain regions should have identical transcriptional signatures. The SVZ region, in close proximity to the brain ventricles, is known to contain specialized niche vasculature. The resulting Harmony-inspired batch correction placed dentate NSCs into clusters with SVZ NSCs, in which some SVZ NSCs are more closely related to dentate NSCs, than SVZ NSCs are to each other. This analysis outcome is not supported by biological evidence, and calls into question the workflow of incorporating this Harmony-inspired batch correction upstream, and the subsequent downstream analyses and results.

3) It is now well established that postnatal and adult murine NSCs produce distinct progeny. The dentate NSCs generate glutamatergic hippocampal neurons, while the SVZ NSCs give rise to several classes of interneurons in the olfactory bulb, with the predominant population being GABAergic. The transcriptional programs determining glutamatergic vs. GABAergic neuronal production have been demonstrated to be cell-intrinsically controlled, and successfully utilized for iN studies. Likewise, the distinct progeny fates from dentate vs. SVZ NSCs are intrinsically controlled, but the exact transcription factor combinations and temporal sequences are not fully understood. Thus, the authors could have mined the scRNA seq datasets to explore these important unknowns.

4) To my knowledge, there is no direct experimental evidence via modern lineage-tracing techniques showing that dentate NSCs transplanted into the SVZ can give rise to OB interneuron progeny, and vice versa. Understanding these hardwired programs and the steps necessary to reverse them are important problems to tackle in neural plasticity and repair. These cellular progeny differences are unlikely to be neurogenic niche-dependent, as the authors suggested through their scRNA seq analyses. The existing biological evidence does not support the authors' claim.

5) The definition of adult niche astrocytes is unclear. Dormant astrocytes within the niche may be quiescent NSCs, and retain neural progenitor capabilities when called upon.

Due to these significant problems with the approach, and the lack of any biological validation from analyzing the scRNA seq datasets, the scope of investigation here is limited here. I am not confident about the validity of these results and do not recommend publication
